# Cirrhotic-extracellular matrix attenuates aPD-1 treatment response by initiating immunosuppressive neutrophil extracellular traps formation in hepatocellular carcinoma

**DOI:** 10.1186/s40164-024-00476-9

**Published:** 2024-02-22

**Authors:** Xiao-Tian Shen, Sun-Zhe Xie, Xin Zheng, Tian-Tian Zou, Bei-Yuan Hu, Jing Xu, Lu Liu, Yun-Feng Xu, Xu-Feng Wang, Hao Wang, Shun Wang, Le Zhu, Kang-Kang Yu, Wen-Wei Zhu, Lu Lu, Ju-Bo Zhang, Jin-Hong Chen, Qiong-Zhu Dong, Lu-Yu Yang, Lun-Xiu Qin

**Affiliations:** 1 Department of General Surgery, Huashan Hospital, Fudan University, 12 Urumqi Road (M), Shanghai, 200040 China; 2 Cancer Metastasis Institute, Fudan University, Shanghai, China; 3 Department of Infection Disease, Huashan Hospital, Fudan University, 12 Urumqi Road (M), Shanghai, 200040 China; 4 Department of Dermatology, Huashan Hospital, Fudan University, 12 Urumqi Road (M), Shanghai, 200040 China

**Keywords:** Hepatocellular carcinoma (HCC), Immune checkpoint inhibitor (ICI), Neutrophil extracellular traps (NETs), Extracellular matrix (ECM), Collagen type I (Col1)

## Abstract

**Background:**

Hepatocellular carcinoma (HCC) is closely associatedwith chronic liver diseases, particularly liver cirrhosis, which has an altered extracellular matrix (ECM) composition. The influence and its mechanism of the cirrhotic-ECM on the response of HCC to immune checkpoint inhibitor (ICI) remains less clarified.

**Methods:**

In silico, proteomic and pathological assessment of alteration of cirrhotic-ECM were applied in clinical cohort. Multiple pre-clinical models with ECM manipulation were used to evaluate cirrhotic-ECM’s effect on ICI treatment. In silico, flow cytometry and IHC were applied to explore how cirrhotic-ECM affect HCC microenvironment. In vitro and in vivo experiments were carried out to identify the mechanism of how cirrhotic-ECM undermined ICI treatment.

**Results:**

We defined “a pro-tumor cirrhotic-ECM” which was featured as the up-regulation of collagen type 1 (Col1). Cirrhotic-ECM/Col1 was closely related to impaired T cell function and limited anti PD-1 (aPD-1) response of HCC patients from the TCGA pan cancer cohort and the authors’ institution, as well as in multiple pre-clinical models. Mechanically, cirrhotic-ECM/Col1 orchestrated an immunosuppressive microenvironment (TME) by triggering Col1-DDR1-NFκB-CXCL8 axis, which initiated neutrophil extracellular traps (NETs) formation to shield HCC cells from attacking T cells and impede approaching T cells. Nilotinib, an inhibitor of DDR1, reversed the neutrophils/NETs dominant TME and efficiently enhanced the response of HCC to aPD-1.

**Conclusions:**

Cirrhotic-ECM modulated a NETs enriched TME in HCC, produced an immune suppressive TME and weakened ICI efficiency. Col1 receptor DDR1 could be a potential target synergically used with ICI to overcome ECM mediated ICI resistance. These provide a mechanical insight and novel strategy to overcome the ICI resistance of HCC.

**Supplementary Information:**

The online version contains supplementary material available at 10.1186/s40164-024-00476-9.

## Background

Hepatocellular carcinoma (HCC) is one of the major causes of cancer-related mortality worldwide [1]. Various systemic therapy options have emerged for advanced HCC during the past decade. Immunotherapy using immune checkpoint inhibitors (ICI) has proven to be effective in eliminating solid malignancies including HCC. However, the response rate of HCC to ICI treatment is limited [2, 3]. Liver is considered an immune-exempt organ, and the development and progression of HCC are often accompanied by complex liver disease background that can impair immune response and impact the efficacy of ICI treatment. Therefore, there is an urgent need to identify HCC patients with specific liver diseases that may cause unmet responses to ICI treatment and ultimately improve its efficiency.

HCC is closely associated with cirrhosis, a pathological process characterized by extracellular matrix (ECM) remodeling in the liver [4, 5]. ECM remodeling involves the dynamic changes of its components, which include collagen, glycoproteins, and proteoglycans. Dependent on tumor types, multiple ECM components exert varied effects on ICI treatment with different underlying mechanisms, including interference on antigen presence, exclusion of activated T and NK cells and recruitment of immune-suppressive cells [4, 6]. ECM components facilitate ICI resistance and remain targetable to enhance ICI in bladder, skin, pancreas, and lung cancers [7–9]. Despite the well-recognized role of cirrhosis in the development and progression of HCC, the interactions between altered ECM in cirrhosis (cirrhotic-ECM) and HCC cells, as well as the tumor micro-environment (TME) and their influence on HCC ICI, have yet to be clarified.

Neutrophils are the most abundant immune cells. Metabolic, chemokine and matrix alteration of neutrophils have been identified in HCC TME with multiple functions including secreting pro-inflammatory mediators, regulating immune-activation [10, 11]. Our previous study revealed neutrophils in HCC formed increased extracellular traps (NETs), a net like structure formed by extrusion of DNA and proteins, to trap disseminated HCC cells and promote metastatic capacity of trapped cells [12]. In addition, NETs may inhibit the cytotoxicity of T and NK cells and induce pro-tumor Th17 signaling to impair immune activation [13–15]. However, how neutrophils and NETs are accumulated in the TME of HCC, especially in cirrhotic-ECM, and their influence on ICI treatment require further exploration.

In this study, we uncovered a Col1-dominant cirrhotic-ECM profile in HCC using muti-omics data with systemic bio-informatics analysis. Using various clinical cohort and pre-clinical models, we found that cirrhotic-ECM and Col1 orchestrated a NETs-enriched immune-suppressive TME, thus impaired aPD-1 efficiency. Mechanically, cirrhotic-ECM recruited neutrophils and initiated NETs by provoking tumorous CXCL8 through Col1-DDR1-NFκB axis. Modulating cirrhotic-ECM by targeting DDR1 with nilotinib efficiently reverse NETs-dominant immune-suppressive TME, enhancing the aPD-1 treatment in HCC with cirrhosis, presenting an appealing therapeutic prospect.

## Material and method

### Human specimens, animal models, cell lines and public data resources

Tumor or normal liver tissues, and peripheral blood samples were obtained from the patients who underwent liver resections for HCC, or benign liver lesions such as hemangioma and focal nodule hyperplasia (FNH) in our institute with the informed consent of the patients. Tissue samples were preserved by frozen or fixed in 10% formalin. These were approved by the Research Ethics Committee of Huashan Hospital, Fudan University (KY2023-594, Shanghai, China).

5–8 weeks male C57BL/6 or nude mice were purchased from Gempharmatech Co., Ltd (Nanjing, China. https://www.gempharmatech.com/) and kept in a SPF (specific pathogen free) room at 25 °C, 50–60% moisture. Human HCC cell line HepG2, PLC/PRF/5(refer to PLC below), Huh7 and murine HCC cell line Hepa1-6 were obtained from National Collection of Authenticated Cell Cultures (Shanghai, China. https://www.cellbank.org.cn/). All human and mouse liver cancer cells were cultured in DMEM with 10% FBS, glutamine and 1% penicillin–streptomycin (Gibco) at 37 °C and 5% CO_2_. Public data resources included transcriptional data of TCGA-LIHC and mass spectrometry proteomics data of CHCC-HBV (Chinese HCC patients with HBV infection), which were downloaded from GDC Data Portal (https://portal.gdc.cancer.gov/) or from additional table S1 from ref [16].

### ECM preparation and nanoscale liquid chromatography coupled to tandem mass spectrometry (nanoLC-MS/MS) analysis

To prepare ECM, fresh human or murine HCC samples were collected and processed within 2 h. About 1 cm^3^ tissue was frozen and sliced into 100 μm slices using a cryotome. Around 100 pieces of sectioned tissue was collected in a 50 ml conical tube and washed with 1: 0.9% saline overnight to remove plasma, 2: 1% SDS for 48–72 h to completely remove cellular component and 3: PBS for 24 h to completely remove SDS. 4: 1 mg/ml DNAse (10607ES15, Yeasen company) for 2 h to remove DNA. In some set of experiment, we depleted Col1 from ECM with collagenase type I (YEASEN) for 10 min at 37 °C. The deposition was collected by centrifuge for 10 min, 3000 rpm, and then resuspended in PBS (referred to as Col1-depleted ECM, or ECM-Col1). ECM or ECM-Col1 was lyophilizated and grinded into powder using 0.5 mm mill beads with a homogenizer.

For cell-culture or in vivo experiment, around 10 mg ECM powder was digested with pepsin in HCL solution (PH = 1), then neutralized with NaOH and diluted into 3 mg/ml. for nanoLC-MS/MS analysis, 10 mg ECM powder was consumed and the analysis was performed at biotree company (www.biotree.cn). Raw data was provided in Additional file 16: Table S4.

### Establishment of mouse models

For ECM-altered orthotopic HCC murine model, 5 weeks old male C57BL/6 mice were intraperitoneal injected with 10 μl/kg dimethyl nitrosamine (DMN, No. 1466674 Sigma) 3 times a week for 4 weeks to develop cirrhosis. Then 1 × 10^6^ Hepa1-6 cells were suspended with 20 μl Matrigel (No. 356234 Corning) and injected under the liver capsule to form HCC.

For ECM altered subcutaneous HCC murine model, 1 × 10^6^ Hepa1-6 cells was injected subcutaneously into the flanks of 6 weeks old mice. After 1 week when tumor size reached 100 mm^3^, 100 μl of 10 mg/ml ECM solution or 3 mg/ml collagen type I solution (No. 354236 Corning) was intra-tumor injected 2 times a week to enable ECM or collagen deposition. Mice were sacrificed before tumor size reached 1.5 cm^3^.

For genetic engineered HCC murine model, performed by hydrodynamic injection (HDI) of 12.5 μg pT3-EF1a-myrAKT1-HA, 12.5 μg pT-Caggs-NRasG12V and 5 μg pCMV-SB. A volume of plasmid solution equal to 10% of the body weight in sterile Ringer’s solution was injected via the tail vein within 5–7 s [17].

For mice undergo aPD-1 or nilotinib treatment, aPD-1 (No. BE0146, inVivoMab) was administrated by intraperitoneal injection (10 mg/kg) twice a week. Nilotinib (Novartis) was administrated by gavage (100 mg/kg) daily. GSK484 (HY-100514, MCE) was administrated by intraperitoneal injection (5 mg/kg) 3 times a week. IgG (10 mg/kg) or corn oil was used as control.

Tumor volume was determined using the modified ellipsoidal formula, tumor volume = 1/2 length * width^2^, based on digital calipers.

### Quantification of growth supportive ability of Col1/ECM on tumor receiving ICI challenge

To reflect the growth supportive ability of ECM component on subcutaneous implanted tumors, we defined a weight ratio C:

$$C = \frac{{Weight^{component1} }}{{Weight^{component2} }},$$

where Weight^component1^ is the weight of tumor injected with ECM component1, such as ECM or Col1, Weight^component2^ refers to the weight of tumor injected with ECM component2, such as ECM-Col1 or PBS. Weight^component1^ and Weight^component2^ are comparable, as both 2 tumors are implanted on the two flanks of the same mice. Higher C value indicate component1 supports tumor growth better than component2.

### Immunohistochemical (IHC) staining

For IHC, 4% paraformaldehyde or 10% formalin fixed, paraffin-embedded sections were baked at 60 °C for 1 h, deparaffinized in xylene, and rehydrated in a graded series of ethanol solutions. Antigens were unmasked by microwave heating the samples in 10 mmol/l sodium citrate buffer (pH 6.0) for 15 min (5 min, 3 times), and the reaction was quenched using hydrogen peroxide 3%. After washing with PBS, the samples were incubated with the following primary antibodies overnight at 4 °C: anti-MPO (No. 14569, CST), anti-CD3 (No. 85061, CST), anti-H3Cit (No. ab219407, abcam), anti-CXCL8 (No. 94407, CST), anti-COL1A1 (No. 72026, CST). Diaminobenzidine (DAB) was used as a detection system. Quantification analyses were performed using ImageJ software based on the percentage of positively stained cells and the staining intensity per field in representative sections.

### Immunofluorescence assay (IF)

For IF, 4% paraformaldehyde fixed cells seeded in the wells of glass-bottomed dishes or paraffin-embedded sections of patient/mouse tumors were rinsed twice with PBS. The following primary antibodies were used: anti-MPO (No. 14569, CST), anti-CD3 (No. 85061, CST), anti-H3Cit (No. ab219407, abcam). The secondary antibodies Alexa Fluor 488 [Alexa Fluor 488-Labeled Goat Anti-Mouse IgG, Beyotime Biotechnology] and Alexa Fluor 555 (Alexa Fluor 555-Labeled Donkey Anti-Rabbit IgG, Beyotime Biotechnology) were used. Nuclei were counterstained with 4′,6-diamidino-2-phenylindole (DAPI) before imaging. A Leica confocal microscope was used to capture the images. Quantification analyses were performed using ImageJ software based on the fluorescence intensity per cell in representative sections.

### Neutrophils and T cells preparation

Peripheral neutrophils were isolated from HCC patients and healthy donors by a widely used one-step gradient centrifugation method using Polymorphprep (Axis-Shield, Dundee, UK) according to the instruction and maintained in RPMI 1640 supplemented with 5% fetal bovine serum (FBS) for immediate use. T cells were isolated from the peripheral blood mononuclear cell (PBMC) with a CD8+ T Cell Isolation Kit (Miltenyi) according to the instruction and maintained in RPMI 1640 supplemented with 5% FBS for immediate use.

### NETs preparation

Peripheral human neutrophils were seeded in 6-well plates (1 × 10^7^/well and stimulated with 20 nM PMA (phorbol 12-myristate 13-acetate) for 4 h. Next, without disturbing the NETs, the supernatants were carefully removed using slow suction, and the wells were washed twice to eliminate residual PMA or NET unassociated substances. To digest the NETs, RPMI (1 ml) containing MNase (1 U/ml) was then added followed by incubation at 37 °C for 20 min. The nuclease activity was then stopped via treatment with 5 mM EDTA. The NET-containing supernatant was collected and centrifuged to eliminate cellular debris. The isolated NETs were stored at − 80 °C for future use. The DNA concentration was applied as NETs concentration.

### NETs formation and cytotoxicity assays

1 × 10^5^ HepG2 cells were seeded in 12-well plate coated with 0.5 ml of ECM (10 mg/ml), ECM-Col1 (10 mg/ml), Col1 (1 mg/ml) or PBS, after formation of small clonality, peripheral neutrophils from HCC patients were co-cultured with tumor cells at 20:1 ratio for 4 h for allowance to extrude NETs. Then neutrophils were gently removed by PBS washing, leaving tumor cells covered by different amounts of NETs. Then 2 × 10^6^ aCD3 plus aCD28 (Biolegend) activated CD8+ T cells from PBMC were added to the culture assay and co-cultured for 2 days, to test how NETs affect T cells cytotoxicity.

Tumor cell survival was examined by flow cytometer. After co-cultured with T cells, suspended immune cells were gently removed with PBS. Then cells were collected with Trypsin and labeled with CSFE (Thermo) and then fixed with 4% paraformaldehyde. Then anti-CD45 antibody (BD-562886, BD) was used to mark immune cells in assay. Then flow cytometer was used to detect tumor cell survival rate. Live tumor cells were marked as CD45^−^CSFE^high^

Confocal speculation was performed 10 h after T cell address. Tumor cells and T cells were marked with ER tracker blue (E12353, Thermo) or DiO (D3911, Thermo) before added into co-culture assay. NETs were marked with SytoxGreen (S7020, Invitrogen).

### Vectors and cell transfections

An expression vector mediated by lentivirus for human DDR1 was constructed. DDR1 cDNA clone (NM-001954) was inserted into pCDH-puro expression vector (System Biosciences). In addition, 2 short hairpin RNAs (shRNA) targeting the human genes DDR1 were synthesized using the primers listed in Additional file 15: Table S3. All these constructs and oligonucleotides were transfected into HCC cells using Lipofectamine 2000 according to the product manual (Invitrogen).

### Construction of ECM score

FPKM-normalized RNA-seq expression counts were downloaded from UCSC Xena (http://xena.ucsc.edu/). A list of reference ECM genes was derived from matrisomeDB (Additional file 14: Table S2). The ECM score, which was used to quantify the enrichment of ECM in tumors, was calculated via gene set variation analysis (GSVA) with the GSVA package. This analysis is based on a non-parametric and unsupervised method that is commonly used to estimate variation in the activities of pathways and biological processes in samples from expression datasets. The ECM scores were grouped by quantiles into 4 groups for categorization and further analysis.

### Neutrophil and T cell chemotaxis assay

Neutrophil chemotaxis was assayed in a Transwell system using 5-μm polycarbonate membrane. Briefly, 1 × 10^5^ HepG2 cells were seeded on the lower chamber of Transwell assay, then 1 × 10^6^ neutrophils were loaded on the upper chamber, followed by incubation for 2 h. Neutrophils that migrated to the lower chamber were collected and counted in Neubauer chambers.

To assess the effect of ECM/Collagen I on T cell chemotaxis, 10 μl of ECM (1 mg/ml), ECM-Col1 (1 mg/ml), or NETs (100 μg/ml) was loaded on the membrane of Transwell chamber. Total fluid volume was controlled at 20 μl with PBS. 1 × 10^5^ T cells were loaded on the upper chamber for chemotaxis, and 500 μl HepG2 conditional media was loaded on the lower chamber to induce chemotaxis for 4 h. Flow cytometer was used to count the T cell number in the lower chamber.

### RNA isolation, reverse transcription, and qPCR

RNA was isolated from cell lines using Trizol reagent (Invitrogen). RNA was quantified using a Nanodrop ND-1000 (Thermo Fisher Scientific). Complementary DNA synthesis was performed using the PrimeScript RT Reagent Kit (Takara) according to the manufacturer’s directions. Real-time PCR was performed using SYBR Green (Takara) and an ABI PRISM 7900 Sequence Detection System (Thermo Fisher). The results were normalized to GADPH for mRNA measurement. Fold change was calculated by the 2ΔDDCt method where DDCt ¼ DCt (Target-Reference) Treatment – DCt (Target-Reference) control. All the primers are listed in Additional file 15: Table S3. qPCR was conducted three times with three repetitions.

### ELISA

Cell medium was collected for detection of CXCL8. An Elisa kit (RK00011, abclonal) was used and experiment was carried out according to the instruction.

### Western blotting

Total protein was extracted by lysing cells in RIPA buffer containing protease inhibitor. Protein samples were separated by SDS-PAGE and transferred onto polyvinylidene fluoride membranes. After blocking with 5% nonfat milk in TBS-T, membranes were incubated with primary antibody. The following antibodies were used: anti-DDR1 (#3917, CST) and anti-p-DDR1 (Tyr513) (#14531, CST), anti-GAPDH (#2118, CST), anti-CXCL8 (#94407, CST), anti-p65 (#8242, CST), anti-pp65 (ser536) (#3033, CST), anti-COL1A1 (#72026, CST). Protein bands were detected by image acquisition using an ImageQuant LAS 4000 (GE Healthcare Life Sciences).

### Flow cytometer

Flow cytometer was used to detected T cell cytotoxicity, T cell and neutrophil fraction in tissue or as cell counting. For culturing cells, cells were digested with Trypsin followed by antibody marking. For tissue sample, cells were pre-digested with collagenase till in single cell state, then followed by antibody marking. Antibody used in this manuscript include CD8-FITC (#140403, biolegend), CD45-BV605 (#013155, biolegend), CD3-PE-Cy7 (#100219, biolegend), Ly6g-FITC (#127605, biolegend), Gr1-Per/Cy5 (#108427, biolegend) and CD11b-PE (#101207, biolegend).

For T cell toxicity assay, T cells were stimulated with 1 μl of cell activation cocktail (#423303, biolegend) for 4.5 to 6 h at 37 °C. After stimulation, cells were fixed and permeabilized using fix/perm working solution (562574, BD) and intracellular staining permeabilization wash buffer (562574, BD) according to the manufacturer’s protocol. Intracellular staining was performed using aIFN-γ and aGZMB (biolegend) antibodies. Flow cytometry data acquisition was performed using a flow cytometer instrument (CytoFLEX). Flow cytometry data were analyzed using Flowjo (v10.8.1) software.

### Statistical analysis

Statistical analyses were performed using the R 4.0.4 software. Student t test and one- or two-way ANOVA were used for comparisons between groups. For all tests, significance was determined with a 95% confidence interval (ns, P > 0.05; *P < 0.05; **P < 0.01; ***P < 0.001; ****P < 0.0001).

## Results

### ECM alteration in HCC with liver cirrhosis was characterized by Col1 up-regulation

**Fig. 1:**
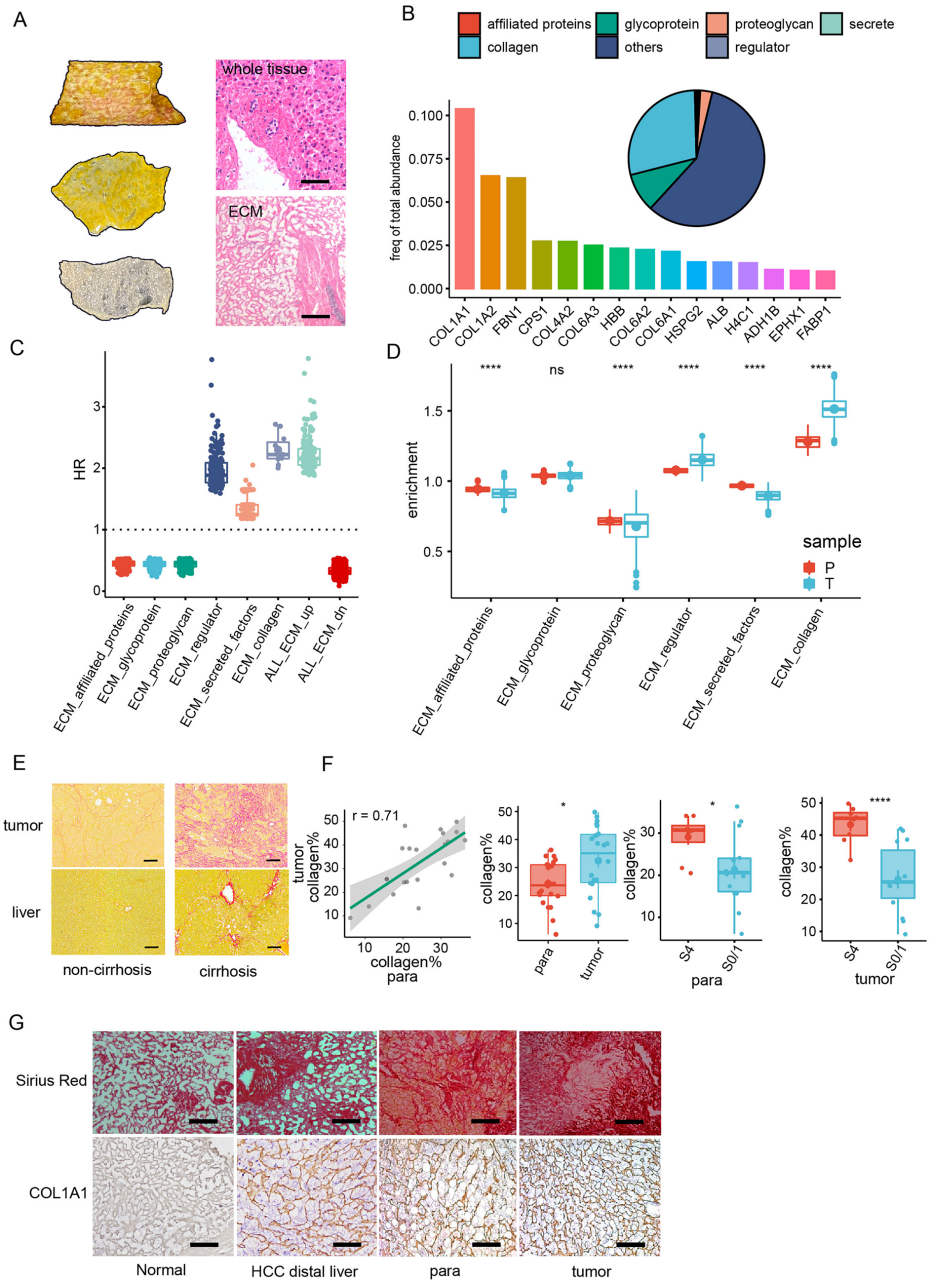
Cirrhotic-ECM alteration in HCC was characterized by Col1 up-regulation. **A** 3 steps de-cellularization process to isolate cirrhotic-ECM from HCC specimens. Gross appear (left) and HE staining (right) showed a complete loss of cellular composition. Scale Bar: 100 μm. **B** Label free quantitative proteomics analysis of the abundance and component of cirrhotic-ECM in HCC. **C** Cox regression showed the Hazard Ratio (HR) of ECM genes in TCGA LIHC cohort. ECM genes are categorized according to matrisome. ECM_up/dn referred to the up-regulated or down-regulated ECM genes in HCC compared with para-tumor tissue. **D** GSEA enrichment score (NES) of ECM genes between para- and tumor-tissue in TCGA-LIHC cohort. **E**, **F** Sirius red staining of collagen fiber composition in HCC para- and tumor-tissue with or without cirrhosis (**E**), and quantification (**F**). Scale Bar: 100 μm. Collagen% referred to the area percentage of collagen fiber. Cirrhosis stage is categorized from S0 to S4 according to the pathological grading and staging systems for chronic hepatitis. **G** Collagen deposition in ECM from normal liver, HCC distal liver tissue, HCC para- and tumor-tissue by Sirius red and IHC staining of COL1A1. Scale Bar: 100 μm. *P < 0.05, **P < 0.01, ***P < 0.001, ****P < 0.0001

To obtain a comprehensive view of cirrhotic-ECM, we first collected ECM structure from freshly resected human HCC tissue with advanced background liver fibrosis (hereafter referred to as cirrhotic-ECM) by performing a 3-step washout of plasma and cellular components followed by H&E staining confirmation (Fig. 1A). We then utilized a label-free quantitative proteomics approach to identify the components present in cirrhotic-ECM. Approximately 45% of the HCC cirrhotic-ECM enrichment fraction belonged to matrisomeDB, a collection of genes that encode core ECM proteins such as glycoproteins, collagens, and proteoglycans, as well as ECM-associated proteins [18]. Among them, CoI1 encoded by COL1A1 and COL1A2 ranked top, with each accounting for 10% and 6% of the total ECM protein, respectively (Fig. 1B).

To further explore the role of cirrhotic-ECM and its key component Col1 in HCC, we analyzed several cirrhotic-ECM encoding genes categorized by matrisome component in TCGA-LIHC cohort (Additional file 1: Fig. S1A), and found among them the up-regulated collagen genes were most closely related to worse clinical outcomes [18] (Fig. 1C). GSEA analysis confirmed collagen was also the most significantly enriched ECM component between HCC tumor and peri-tumor liver region (Fig. 1D). A matrix score based on matrisome genes was established, and it was found to be higher expressed in HCC with cirrhosis, and was related to poor survival (Additional file 1: Fig. S1B–D). Of note, certain matrisome components, including affiliated proteins and glycoprotein, displayed diverse regulatory patterns and optical effects on prognosis, indicating the complex role of cirrhotic-ECM in HCC. Using Sirius red staining, we demonstrated a correlated distribution of collagen levels in both non-tumor liver and tumor regions. In HCC with advanced cirrhosis (fibrosis stage 4), collagen deposition was significantly higher in both para-tumor and tumor regions (Fig. 1E, F). We also observed a stepwise increasing deposition of collagen, especially Col1, in ECM from normal liver, non-tumor cirrhotic liver, cirrhotic peri-tumor liver to HCC tissues by Sirius red staining and IHC staining of COL1A1 (Fig. 1G). Clinically, whole ECM or collagen composition was a predictor of a worse survival for HCC and was relevant to clinical parameters, such as increased tumor marker, solitary tumor, encapsulation, tumor thrombosis, higher recurrence or metastasis rate in our HCC cohort (Additional file 13: Table S1).

Taken together, these results indicated that cirrhotic-ECM which is featured by Col1 up-regulation is closely associated with and can be a predictor for a dismal clinical outcome of HCC.

### Cirrhotic-ECM and Col1 attenuated aPD-1 therapy efficiency by interrupting T cell function in HCC

**Fig. 2:**
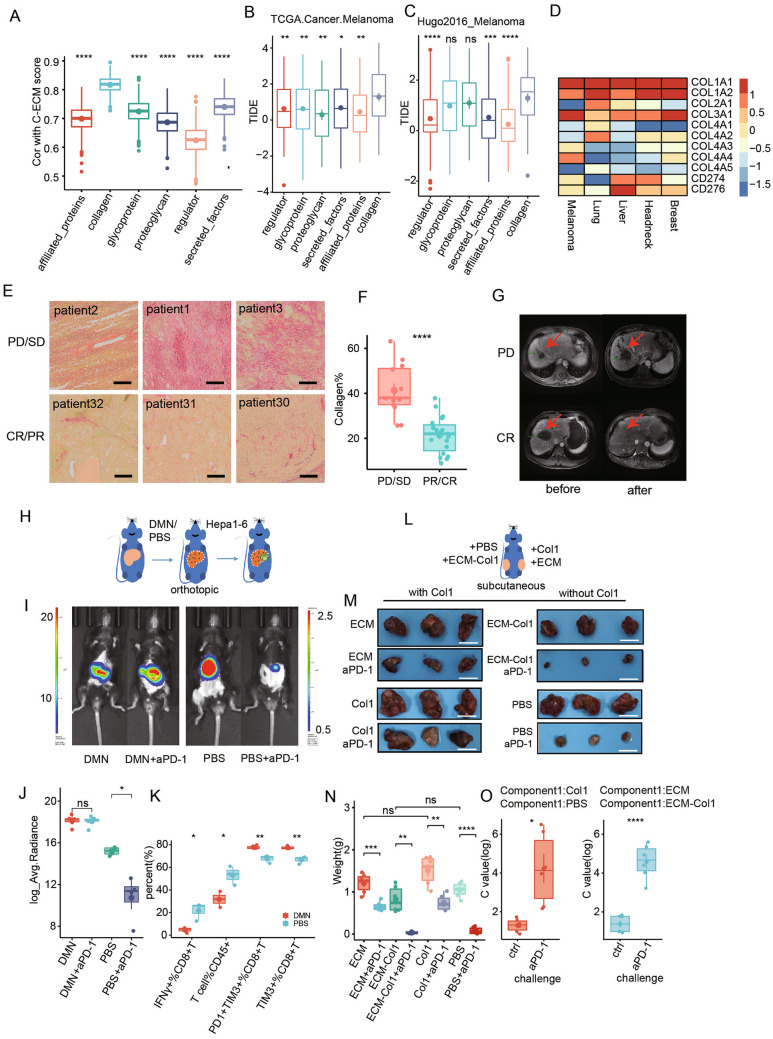
Cirrhotic-ECM and Col1 attenuated aPD-1 therapy by interrupting T cell cytotoxicity in clinical cohorts and pre-clinical models. **A** Correlation between GSEA enrichment score of ECM composition and C-ECM score in TCGA LIHC cohort. **B**, **C** TIDE score and ECM composition enrichment in 2 melanoma cohort treated with aPD-1 in TCGA cohort. **D** Heatmap of pan-cancerous correlation between TIDE score and collagen genes. **E**, **F** Sirius red staining of collagen fiber in aPD-1-treated HCC patients with well (n = 19) or poor responses (n = 12). Scale Bar: 500 μm. **G** Representative MRI images of the different response HCC patients to aPD-1 treatment. **H** Establishment scheme of Hepa1-6 orthotopic implantation HCC model in C57BL/6 mice w/o cirrhotic-ECM alteration. **I**, **J** Representative bioluminescence images of tumors w/o aPD-1 treatment in orthotopic implantation HCC model w/o ECM alteration (**I**), and quantification of average radiance of fluoresce intensity of photon flux (**J**). **K** Quantification of Intratumor IFNγ+ T cell, PD-1+TIM3+ T cell and TIM3+ T cell across subgroups of orthotopic implantation HCC model w/o cirrhotic-ECM alteration by flow cytometry. **L** Establishment scheme of Hepa1-6 subcutaneous implantation HCC model in C57BL/6 mice w/o cirrhotic-ECM/Col1 alteration. **M**, **N** Images and quantification of tumor w/o aPD-1 treatment in subcutaneous implantation HCC model w/o cirrhotic-ECM/Col1 alteration. Scale Bar: 1 cm. **O** C value of ECM or Col1 under aPD-1 or control treatment challenge. X axis showed whether tumor underwent aPD-1 treatment challenge. *P < 0.05, **P < 0.01, ***P < 0.001, ****P < 0.0001

One intriguing question is whether cirrhotic-ECM impacts the response to ICI therapy in HCC. To investigate this, we first employed a bioinformatics approach and utilized the C-ECM score, a scoring system based on ECM genes that predicts ICI response. Collagen was demonstrated the strongest correlation with the C-ECM score among all the key ECM components in the TCGA-LIHC cohort (Fig. 2A) [19]. Given the lack of ICI response data in TCGA-LIHC cohort, we assessed ECM components’ effect on ICI using melanoma and bladder cancer cohort. To be noted, higher Tumor Immune Dysfunction and Exclusion (TIDE) score indicates such component is more responsible for ICI resistance. As expected, collagen had the highest TIDE score among all ECM components (Fig. 2B, C). Thus, we expanded the TIDE score analysis to dataset of HCC and other solid tumors, and confirmed collagen type I, among other collagens, has the highest TIDE score, even higher than classical T cell suppress protein PD-L1 and PD-L2. These findings suggest that collagen type I may function to suppress T cell activity (Fig. 2D).

To evaluate the potential impact of cirrhotic-ECM and collagen on the response of HCC to ICI, we gathered data from the advanced HCC patients who received aPD-1 treatment and had available response data at our institute. We measured the level of tumorous collagen and found that, consistent with our bioinformatics exploration, HCC patients who exhibited a poor response to aPD-1 treatment (PD or SD by RECIST criteria) had a higher collagen level compared with those who a better treatment response (CR or PR) (Fig. 2E–G).

To further investigate the potential impact of cirrhotic-ECM on aPD-1 treatment, we established the ECM-enriched model by inducing cirrhosis in immune-competent mice using DMN, and then established orthotopic and subcutaneous implantation HCC models using Hepa1-6 cells. Consistent with the findings in clinical cohorts, DMN-induced cirrhosis promoted tumor progression in the orthotopic implantation model. Treatment with aPD-1, which proved effective in non-cirrhotic mice, failed to exert tumor inhibitory effect in the DMN-induced cirrhotic model (Fig. 2H–J). Flow cytometry analysis demonstrated a less immune-activation (IFN-γ, GZMB) and a higher immune-exhaustion (PD-1, TIM3) indicator in tumor-infiltrating T cells from HCC with ECM-enriched cirrhosis (Fig. 2K). Furthermore, we extracted ECM components from the HCC tissue from the mice model with DMN-induced cirrhosis (cirrhotic-ECM) and subcutaneous implantation models. Similar to the orthotopic implantation model, these extractions displayed an equivalent pro-tumor ability and inhibitory effect on aPD-1 response. Moreover, Col1 injection alone, which was identified as the key component of cirrhotic-ECM in HCC, exerted a comparable inhibition on aPD-1 efficacy (Fig. 2L, M). ECM or Col1 showed a more significant tumor growth promoting ability (C value) of HCC under aPD-1 treatment compared with controls (Fig. 2N, O, see method). This was companied with a similar reduction of intra-tumor CD8+ T cells and cytotoxicity factor TNFA and GZMB (Additional file 2: Fig. S2). Taken together, these results demonstrated that cirrhotic-ECM and Col1 can attenuate the efficacy of aPD-1 therapy by disrupting T cell cytotoxicity.

### Cirrhotic-ECM and Col1 orchestrated a neutrophils- and NETs-enriched immune-suppressive tumor microenvironment in HCC

**Fig. 3:**
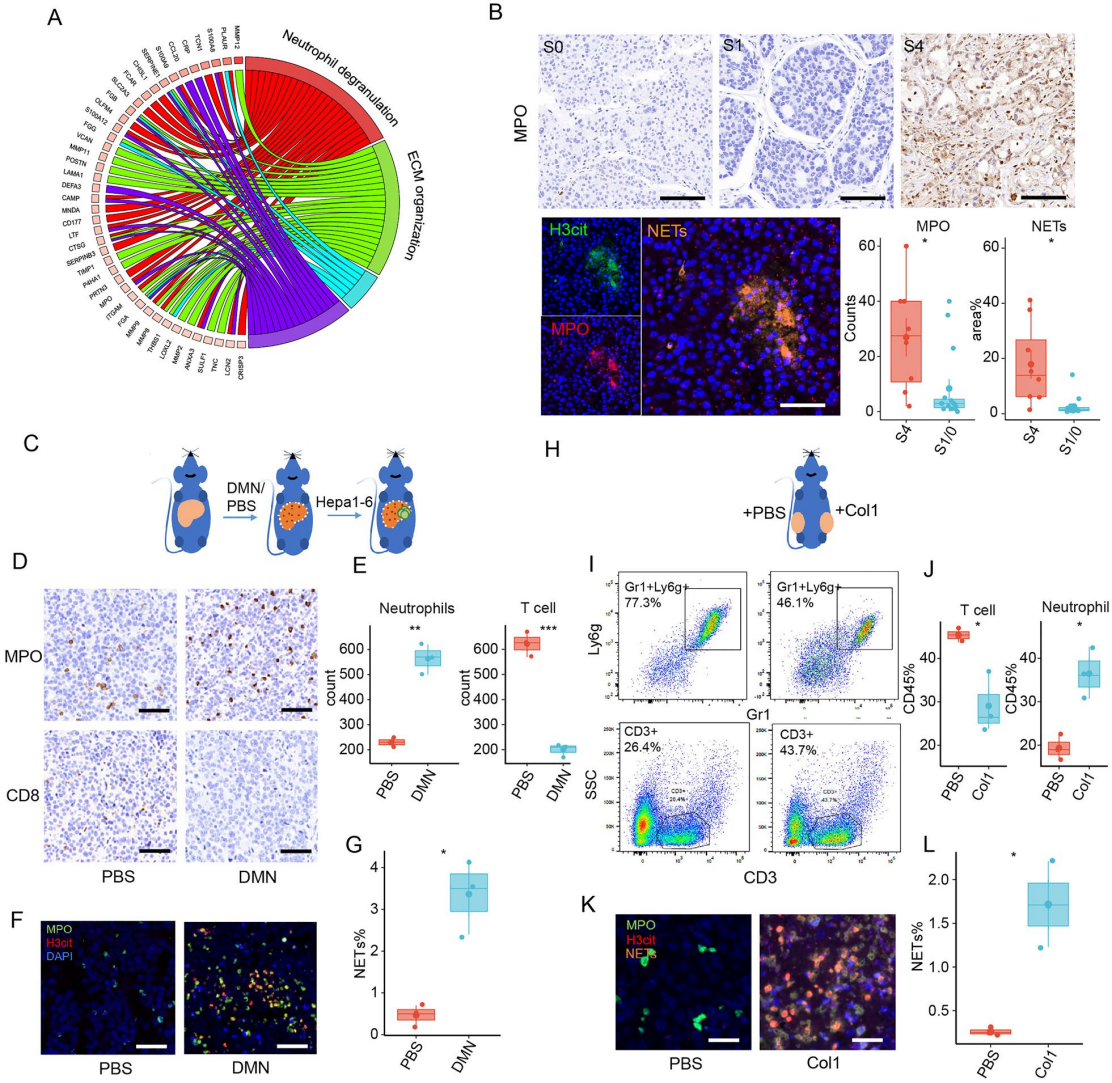
Cirrhotic-ECM orchestrated a neutrophils/NETs-dominant immune-suppressive TME through Col1. **A** Chord plot of KEGG enrichment based on DEGs from patients with ECM score -high and -low in TCGA-LIHC cohort. **B** Representative images and quantification of IHC and multiplex fluorenes staining of MPO and H3cit in HCC patient with fibrosis. Scale Bar: 50 μm. **C** Establishment scheme of Hepa1-6 orthotopic HCC model in C57BL/6 mice w/o DMN-induced cirrhotic-ECM alteration. **D**, **E** Representative IHC staining (**D**) and quantification (**E**) of the MPO (neutrophils) and CD8 (T cells) in orthotopic model in **C**. Scale Bar: 100 μm. **F**, **G** Representative multiplex fluorenes staining (**F**) and quantification (**G**) of the MPO+H3cit (NETs) in orthotopic model in **C**. Scale Bar: 100 μm. **H** Establishment scheme of Hepa1-6 subcutaneous HCC model in C57BL/6 mice w/o Col1 co-injection. **I**, **J** Representative flow cytometry images (**I**) and quantification (**J**) of the Gr1+Ly6zG+ neutrophils and CD3+ T cells in subcutaneous model in **H**. **K**, **L** Representative multiplex fluorenes staining (**K**) and quantification (**L**) of the MPO + H3cit (NETs) in subcutaneous model in **H**. Scale Bar: 20 μm. *P < 0.05, **P < 0.01, ***P < 0.001, ****P < 0.0001

ECM has been acknowledged for its broad immune-regulatory capacity in both inflammation and cancer. To investigate the effect of cirrhotic-ECM on the tumor microenvironment (TME) and ICI response of HCC, we analyzed the differentially expressed genes (DEGs) between ECM score-high and -low subgroups of the TCGA LIHC cohort using the KEGG enrichment method, and revealed that the major difference was in neutrophil function, rather than T cell cytotoxicity or exhaustion (Fig. 3A and Additional file 3: Fig. S3A). Using cell marker gene expression and the Cibersort deconvoluted cell fraction method, a neutrophil-dominant TME was demonstrated in ECM-high HCC at both proteomic (CHCC-HBV cohort) and transcriptional levels (TCGA-LIHC cohort) (Additional file 4: Fig. S4A, B).

Neutrophils within the TME, which are known to form extracellular traps (NETs), have been shown to exert a complex regulatory capacity in many types of cancer. We have also found that increased neutrophils and NETs promote HCC growth and progression by provoking a tumorous inflammatory response [12, 20]. In the present study, the enrichment of NETs was further observed in ECM score-high HCC (Additional file 4: Fig. S4C, D).

Re-analyzing the multi-parameter flow cytometry data of our previous study [21] further validated an increased neutrophils accumulation in HCC with advanced cirrhosis (Additional file 5: Fig. S5). Consistently, immunohistochemistry (IHC) for the neutrophil marker MPO and the NETs marker MPO-H3Cit in an independent HCC cohort with advanced cirrhosis from our institute also demonstrated a higher distribution of neutrophils and NETs (Fig. 3B, Additional file 13: Table S1). In orthotopic transplantation or HDI-induced murine HCC model, we observed that DMN-induced cirrhosis increased neutrophils and NETs in the tumors, with or without a decrease of CD8+ T cells (Fig. 3C–G and Additional file 6: S6), which was consistently observed in the subcutaneous implantation model (Fig. 3H–L). These findings suggested that cirrhotic-ECM and its key component Col1 play a significant role in orchestrating a neutrophil- and NETs-enriched TME in both human and mice.

### Cirrhotic-ECM enriched in Col1 initiated NETs formation to shield tumor cell and impede T cell motivation

**Fig. 4:**
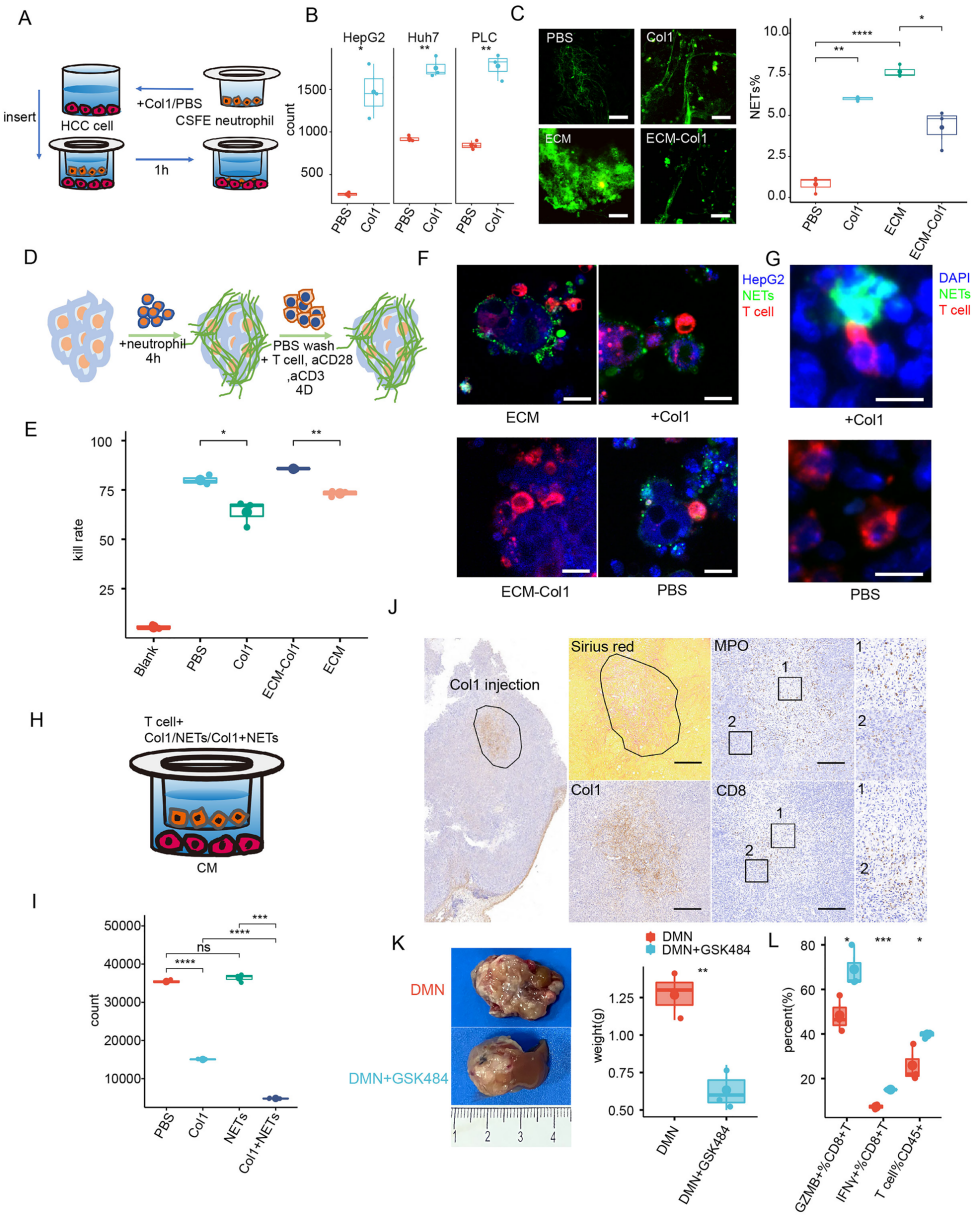
Col1-enriched cirrhotic-ECM initiated neutrophils to form NETs to shield tumor cells and impede T cell motivation. **A**, **B** Establish scheme (**A**) and quantification (**B**) of neutrophils chemotaxis to Col1-treated HCC cells in a Transwell system. Col1 and HCC cell were co-placed on the lower chamber, and CSFE-labeled neutrophils were placed on the upper chamber. **C** Representative images and quantification of SytoxgGreen-marked NETs formation in a co-culture system of neutrophils and HepG2 w/o ECM/ECM-Col/Col1. Scale Bar: 5 μm. **D** Scheme plot of the HepG2-neutrophil-T cell co-culture assay in dishes coated w/o ECM/ECM-Col/Col1. **E** Quantification of HepG2 survival by flow cytometry in assay in **D**. **F** Fluorescence images of NETs (SytoxGreen) shielding HepG2 cell (ER tracker blue) from adherent T cells (Dio red) w/o Col1. Scale Bar: 5 μm. **G** Representative fluorescence images of local spatial distribution of HCC cell (Hoechst), NETs (H3cit, green) and T cell (CD8 red) in Fig. 3H. Scale Bar: 5 μm. **H** Establish scheme (**H**) and quantification (**I**) of T cells chemotaxis to Col1-treated HCC cells in a Transwell system by flow cytometry. HCC cells were placed on the lower chamber of Transwell assay. PET membrane was coated with Col1/NETs/Col1+NETs. CSFE-labeled T cells were placed on the upper chamber. **J** IHC images of spatial distribution of intra-tumor T cell and neutrophils at the intra-tumor injection site of Col1 in Hepa1-6 subcutaneous model. Scale Bar: 200 μm. **K**, **L** Images of Tumor (**K**), and quantification (**L**) of Intra-tumor GZMB+CD8 T cell, IFN-γ+CD8 T cell and CD8 T cell by flow cytometry in Hepa1-6 orthotopic model with DMN-induced cirrhosis w/o GSK484. *P < 0.05, **P < 0.01, ***P < 0.001, ****P < 0.0001

We further assessed the interplay between cirrhotic-ECM/Col1 and neutrophils in TME. Despite Col1 alone failed to increase neutrophils chemotaxis (Additional file 7: Fig. S7), the conditioned medium from the Col1-treated HCC cells showed a significant effect on increasing neutrophil chemotaxis (Fig. 4A, B). Furthermore, both cirrhotic-ECM and Col1, rather than the Col1-depleted cirrhotic-ECM, were observed to increase NETs formation in a co-culture system of HCC cells and neutrophils (Fig. 4C). We further employed an ECM-coated co-culture system consisting of HepG2 cells, neutrophils, and activated CD8+ T cells, which allowed for the simultaneous NETs formation and tumor cell killing (Fig. 4D). As expected, T cells cytotoxicity on HepG2 cells was significantly impeded by cirrhotic-ECM/Col1-iniated NETs, but not Col1-depleted cirrhotic-ECM (Fig. 4E). Under confocal microscopy, we observed massive NETs formed and assembled in a shield-like structure around HepG2 cells, which prevented T cells from contacting and killing tumor cells in the cirrhotic-ECM/Col1-coated condition (Fig. 4F). Such NETs shields were also detected at a higher frequency in the subcutaneous co-implantation tumor with Col1 (Fig. 4G). When NETs shields were removed, the contact of T cells with HepG2 cells was restored (Additional file 8: Fig. S8). In addition to shielding HCC cells from T cells, Transwell assay showed that Col1 mixed with NETs also diminished T cell chemotaxis induced by HCC medium, and thus further inhibited T cell contact (Fig. 4H, I). Moreover, administration of NETs also impeded contacted T cells cytotoxicity by reducing IFN-γ and GZMB secretion (Additional file 9: Fig. S9). Immune-staining revealed abundant local neutrophils and absence of T cells within the central region of the subcutaneous tumors implanted with Col1 (Fig. 4J). Given the above essential role of NETs in impeding T cells function within cirrhotic-ECM, we blocked NETs formation by GSK484 in orthotopic Hepa1-6 mice models with DMN-induced cirrhosis, which resulted in a significant decrease in tumor volume and increase of T cell activation (Fig. 4K, L).

Taken together, these results suggested that neutrophils form more NETs in cirrhotic-ECM/Col1 enriched TME, thus retard distant T cells motivation and shield HCC cells from adherent T cells attacking.

### Col1 activated DDR1-CXCL8 axis to attract neutrophils and promote NETs formation

**Fig. 5:**
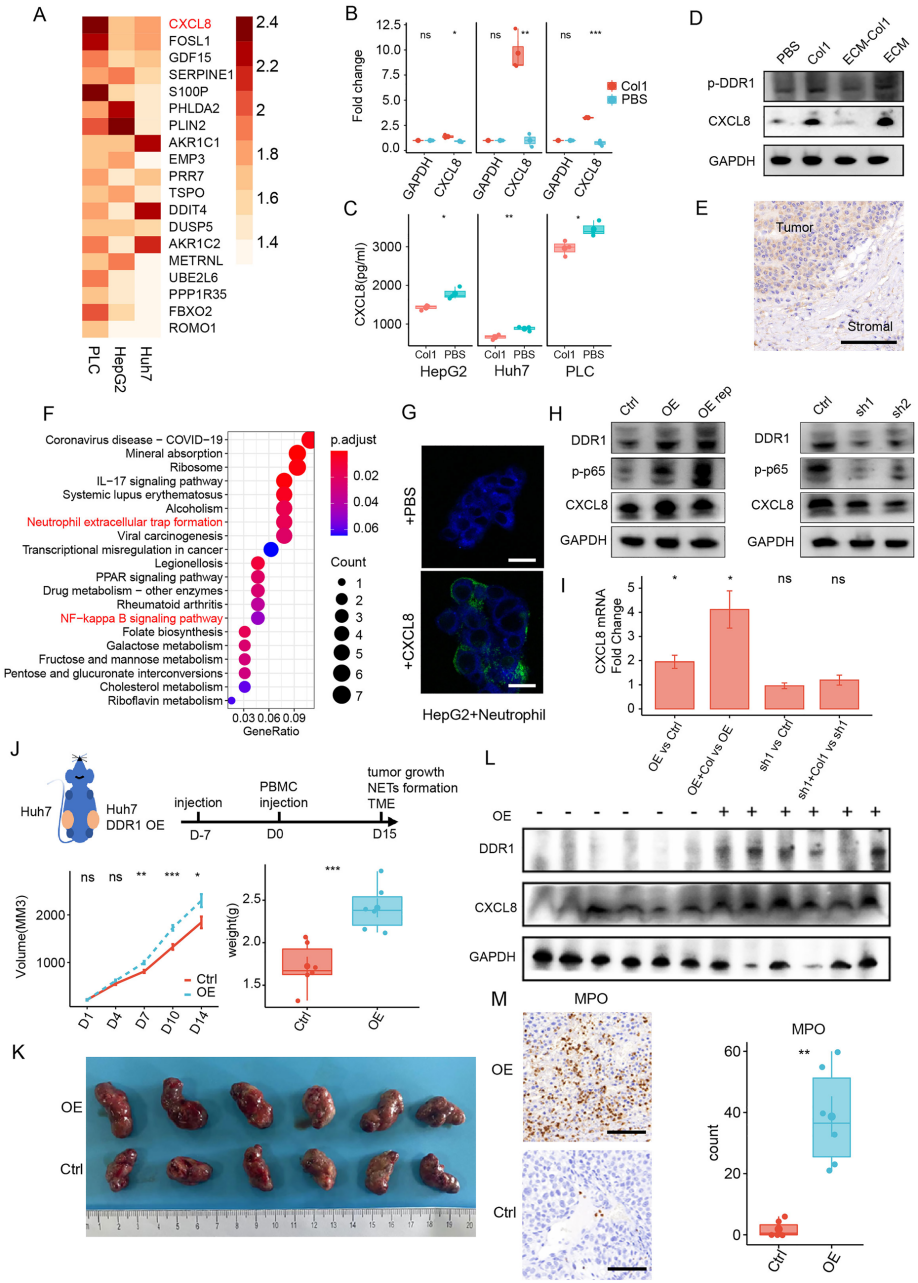
Col1 in cirrhotic-ECM triggered tumorous DDR1-NFκB-CXCL8 axis in HCC to recruit neutrophils/NETs. **A** Heatmap of up-regulated genes in PLC/HepG2/Huh7 treated with cirrhotic-ECM by RNA-seq. **B** CXCL8 mRNA level change in PLC/HepG2/Huh7 treated with Col1 by qPCR. **C** CXCL8 level change in PLC/HepG2/Huh7 treated with Col1 by Elisa. **D** Phosphorating DDR1 and CXCL8 level change in Huh7 treated with Col1/ECM-Col1/ECM by Western Blot. **E** CXCL8 expression in human HCC specimen by IHC. Scale Bar: 50 μm. **F** KEGG annotation of up-regulated genes in **A**. **G** NETs formation by immune-fluorescence staining. Normal human peripheral neutrophils were co-cultured with HepG2 cells w/o exogenous CXCL8, NETs were then fixed and labeled with SytoxGreen. Scale Bar: 4 μm. **H** p-p65 and CXCL8 level change in Huh7 with DDR1 knockdown or over-expression by Western Blot. **I** CXCL8 mRNA level change in Col1-treated Huh7 with DDR1 knockdown or over-expression by qPCR. **J** Scheme plot of humanized mice model. Nude mice were subcutaneously implanted with Huh7 cells w/o DDR1 over-expression, and human PBMC were i.v injected to allow T cell infiltration 7 days later. **K** Images of tumor growth and quantification of Huh7 tumor w/o DDR1 over-expression in humanized mice model. **L** CXCL8 expression in Huh7 tumor w/o DDR1 over-expression in humanized mice model by Western Blot. **M** Quantification of intra-tumor neutrophils density (count per ×40 view) in Huh7 tumor w/o DDR1 over-expression by IHC. Scale Bar: 100 μm. *P < 0.05, **P < 0.01, ***P < 0.001, ****P < 0.0001

Both the cirrhotic-ECM/Col1 and HCC cells were required to form immunosuppressive NETs, which indicates the possible important roles of the interaction between them. RNA-seq was performed in HepG2, Huh7 and PLC/PRF/5 cells treated by cirrhotic-ECM. A total of 1961/18139 up-regulated genes were identified. Among of them, CXCL8, a widely recognized inflammatory mediator involved in regulating neutrophil chemotaxis and function, had the highest significance and consistency in all the three cell lines (Fig. 5A, Additional file 17: Table S5). qPCR, ELISA and immune-blotting assays demonstrated that both cirrhotic-ECM and Col1 could induce CXCL8 up-regulation in HCCs (Fig. 5B–D). Furthermore, an obvious upregulation of CXCL8 secreted by tumor cell was also detected by immunohistochemistry in cirrhotic HCCs (Fig. 5E). KEGG annotation of the top 20 up-regulated genes also demonstrated the important roles of NETs and NFκB pathway (Fig. 5F). Moreover, exogenous CXCL8 could promote NETs formation in vitro (Fig. 5G). And a positive correlation was found between CXCL8 and neutrophils marker FUT4 levels in TCGA LIHC cohort (Additional file 10: Fig. S10). These suggested CXCL8 is a critical mediator between HCC cells and pro-tumor neutrophils/NETs in cirrhotic-ECM enriched TME.

Discoidin domain receptor 1 (DDR1) is a well-known receptor for Col1. Immunoblot analysis revealed that cirrhotic-ECM and Col1 induced DDR1 phosphorylation and subsequent up-regulation of CXCL8 in HepG2 cells, whereas Col1-depleted ECM did not show such effects (Fig. 5C). Furthermore, overexpression or knockdown of DDR1 by lentivirus or shRNA was sufficient to modulate Col1-induced CXCL8 up-regulation in both HepG2 and Huh7 cells, as evidenced by immunoblotting. In addition, a coexistence of p65 phosphorylation with DDR1 and CXCL8 levels was observed, which further supports a role of NFκB pathway in the cirrhotic-ECM/Col1-DDR1-CXCL8 axis (Fig. 5H, I and Additional file 11: S11A–C).

Furthermore, we established a humanized mice model by subcutaneously implantation of Huh7^DDR1OE^ or Huh7^PCDH^ in nude mice, and subsequently adoptively transferred with human PBMCs via intravenous injection (Fig. 5J). DDR1 overexpression promoted tumor growth and increased CXCL8 expression, accompanied by a robust increase in immunosuppressive neutrophils (Fig. 5K–M). In TCGA LIHC cohort, the expression levels of DDR1, CXCL8 and p65 were positively correlated (Additional file 10: Fig. S10). Collectively, these findings suggest that cirrhotic-ECM triggers HCC-derived CXCL8 through the Col1-DDR1-NFκB axis.

### DDR1 inhibitor enhanced aPD-1 response through reversing neutrophils/NETs-dominant immunosuppressive TME in HCC

**Fig. 6:**
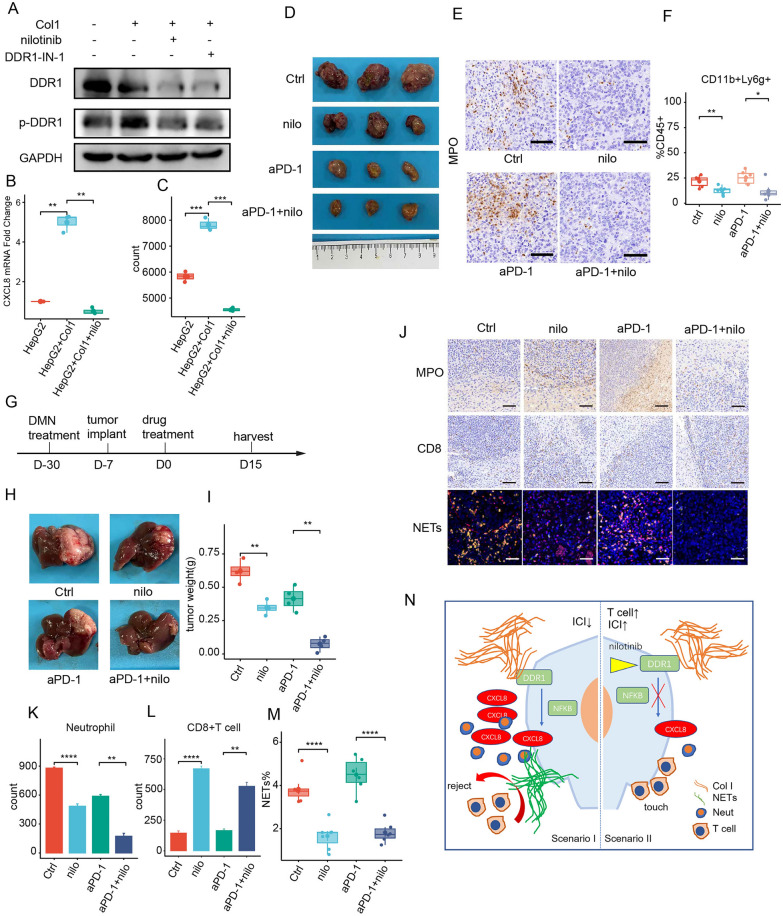
Synergistic effect of inhibiting DDR1 with nilotinib on aPD-1 in cirrhotic HCC through reversing neutrophils/NETs-dominant immunosuppressive TME. **A** Level change of phosphorating DDR1 in Col1-treated HepG2 cells w/o nilotinib and DDR-IN-1 by Western Blot. **B** CXCL8 mRNA level change in Col1-treated HepG2 w/o nilotinib by qPCR. **C** Neutrophils chemotaxis to Col1-treated HepG2 cells w/o nilotinib by flow cytometry in Transwell system. **D**–**F** Tumor growth (**D**), IHC images (**E**) and flow cytometry quantification (**F**) of infiltrated neutrophils in Hepa1-6 subcutaneous mice model challenged with cirrhotic-ECM plus nilotinib (nilo)/aPD-1/nilo+aPD-1. Scale Bar: 100 μm. **G**–**M** Experiment design was shown as **G**. Representative images (**H**) and quantification (**I**) of tumor growth, IHC/IF staining (**J**) and quantification of Ly6G+ neutrophils (**K**), CD8+ T cells (**L**) and NETs (**M**) in Hepa1-6 orthotopic HCC mice model with DMN induced cirrhosis treated with nilotinib (nilo)/aPD-1/nilo+aPD-1. Scale Bar: 100 μm. Cell number was calculated by counts per ×40 view. **N** A schematic diagram described the mechanism of cirrhosis ECM induced NETs performed blockade of T cell cytotoxicity and attenuated ICI efficiency in HCC. *P < 0.05, **P < 0.01, ***P < 0.001, ****P < 0.0001

Nilotinib is a selective inhibitor of DDR1 activation, which has been approved by FDA of USA as an anti-leukemia drug. In vitro studies showed that nilotinib could effectively interrupt Col1-mediated tumorous DDR1 phosphorylation, thus impairing its downstream CXCL8 up-regulation and the increased neutrophil chemotaxis to HCC cells (Fig. 6A–C). We further evaluated the synergistic effect of nilotinib and aPD-1. In either the subcutaneous implantation model with cirrhotic-ECM, or the orthotopic model with DMN-induced liver cirrhosis, nilotinib demonstrated more significant capacities to reduce the immunosuppressive neutrophil infiltration with subsequent reclaimed T cell activation, as well as the inhibitory effect on HCC growth and progression when in combination with aPD-1 compared with its treatment alone (Fig. 6D–F). Importantly, their combination was well tolerated, as there was no significant change in peripheral blood cell count, nephrotic function, or liver function of the tested mice (Additional file 12: Fig. S12). Taken together, these results suggest that nilotinib can reduce the Col1-DDR1-CXCL8-mediated neutrophil-dominant immunosuppressive TME, and thus enhancing the ICI response of HCC.

## Discussion

The excessive condensation of extracellular matrix (ECM) in the tumor microenvironment (TME) plays a critical role in tumor progression and metastasis, as well as therapy resistance through multiple mechanisms. Altered ECM remodeling in cirrhosis further complicates HCC malignancy and treatment. However, a comprehensive analysis of the cirrhotic-ECM alteration profile, as well as the effect of cirrhosis-associated precancerous lesions on HCC TME, remains less clarified. In this study, we performed a bioinformatics analysis of multi-omic HCC cohorts and multiple pre-clinical mice models, combining transcriptional and proteomic sequence data. We identified over-deposition Col1 as the key feature of cirrhotic-ECM in HCC. Consistent with previous reports that collagen or certain ECM components predict a worse survival and represent a phenotype with higher malignancy in several cancers, we further demonstrated a close relationship between cirrhotic-ECM and its key component Col1, and poor prognosis in HCC.

Several mechanisms in the immune modulation of ECM or collagen have been raised. Collagen, by forming dense fiber, can exclude lymphocyte infiltration, which has been observed in various kind of cancers including pancreatic, breast, lung and bladder cancers [22, 23]. Moreover, collagen can regulate cytotoxicity of lymphocyte by binding to collagen receptor, such as LAIR1 or DDR1 [24–26]. These two mechanisms on T cells are prevalent in most type of cancers. Here we demonstrated a novel immune regulating mechanism of cirrhotic ECM that collagen alters tumor cell through DDR1-NFκB-CXCL8 axis, which attracts immune neutrophil/NETs to repress T cell cytotoxicity. Limited report has mentioned this mechanism in other solid malignancies yet.

Although liver cirrhosis is known to contribute to HCC development and progression, limited research has explored how cirrhotic ECM affects immune checkpoint inhibitor (ICI) response in HCC. Despite clinical trials suggest that HCC patients with viral hepatitis, the most common etiology of cirrhosis, may have different ICI responses, direct evidence on cirrhosis and ICI response remains scarce. In line with recent reports, an ECM-based algorithm (C-ECM score) has been found to positively predict ICI response. We provide additional evidence that cirrhotic-ECM/Col1 enrichment is correlated with the TIDE score, a widely accepted ICI predictive indicator. In addition to bioinformatics analyses, we present compelling evidence supporting the detrimental effect of cirrhotic-ECM on ICI response in our HCC cohort, as well as in immune-competent HCC-bearing mice with altered cirrhotic-ECM. This finding is consistent with previous studies in lung and breast cancers, which indicates that collagen promoted ICI resistance through T cell exhaustion or exclusion [26–28]. Importantly, our data suggest that Col1 is an essential component of cirrhotic-ECM for exerting ICI augmentation capacity, as cirrhotic-ECM failed to impair ICI when Col1 was depleted.

The implantation mice models of Hepa1-6 cells are often used in immune therapy study. But they didn’t’ have cirrhotic liver background. DMN-induced cirrhosis and carcinogenesis model might be more suitable to assess how cirrhotic liver background influent HCC response, however, it is a time-consuming and difficult process with a very high mortality of mouse. To deal with this problem, we combined these two models by implanting Hepa1-6 cells into the DMN-induced cirrhotic mice, which could somewhat mimic HCC development and response to treatment in a cirrhotic background.

Reshaped TME composed of Immune-suppressive cells modulating T cells dysfunction is well documented as one key cause of ICI failure. The formation of immune-suppressive TME may result from either tumor-secreted factors or ECM components, but more likely, the interaction between ECM and tumor cells. In this study based on multiple cohorts, we revealed neutrophils to be the most altered immune component in TME of HCC with cirrhosis. The neutrophils recruitment initiated by cirrhotic-ECM required involvement of HCC cells, instead of cirrhotic-ECM or Col1 alone. The transcript analysis revealed cirrhotic-ECM/Col1 acted on neutrophils in an indirect way by triggering HCC cells-secreted key neutrophilic chemokine CXCL8. In HCC, neutrophils accounts for 5–30% of total cell number. Accumulating multi-omics studies at single-cell level uncovered a previous less recognized yet emerging role of neutrophils in either treatment-naïve or treated HCC with prognostic value [11]. Pan-cancerously, multiple mechanisms may be involved in tumor-associated neutrophils to dominate immune-suppressive TME. Under certain stimulation, pro-tumor neutrophils extrude NETs, a web-like structure tightly involved in cancers and inflammatory disorders. Previously we reported increased NETs facilitate HCC metastasis by optimizing tumor seeding and boosting subsequent malignant phenotype [12]. Supported by reports on CXCL8-mediated neutrophils accumulation and NETs formation, here we proved cancerous CXCL8-recruited neutrophils formed increased NETs in cirrhotic-ECM/Col1 enriched TME, which were essential to immune-suppression and poor ICI response.

Role of NETs in impairing ICI has been suggested in pancreatic and colon cancer, where NETs caused immune-suppressive TME by attenuating T cells activation through less known mechanism [15]. In cirrhotic-ECM, we observed the increased NETs together with Col1 wrapped HCC cells and blocked physical contact between tumor cells and cytotoxic T cells, thus impairing ICI response. Moreover, the shield-like structure of NETs and Col1 around HCC cells also trapped and directly exhausted activated T cells through protease and immune-suppressive molecule decorated on chromatin backbone of NETs [29, 30]. In addition to shielding HCC cells from adherent activated T cells, we found cirrhotic-ECM/Col1 and NETs in synergic also ceased distant T cells infiltration into tumor region, thus creating a local neutrophils/NETs-enriched but T cells-deserted region. This is supported by the finding that neutrophils/NETs and T cells were observed oppositely distributed in cirrhotic-ECM/Col1 enriched area, either by us or others [31, 32]. The NETs-enriched and T cell-sparse TME also explains the more aggressive intra-hepatic metastasis in cirrhotic HCC patients.

As one major ECM receptor [33–35], DDR1 pathway played a crucial role in immune regulation, progression, metastasis and carcinogenesis in various types of cancer [33, 34, 36, 37]. Emerging clinical trials highlight targeting DDR1 as a novel systemic strategy. Tyrosine kinase inhibitor nilotinib is a FDA-approved agent for chronic myelogenous leukemia that selectively inhibits DDR1 pathway [38]. Nilotinib, or other DDR1 inhibitors has been proven effective in preclinical cancer models include lung cancer [39], pancreatic cancer [35], ovarian cancer [36] and breast cancer [27]. Here we revealed nilotinib efficiently modulated immune-suppressive TME by blocking Col1-DDR1-CXCL8 axis triggered by cirrhotic-ECM, thus lowing neutrophils/NETs enrichment, reclaiming T cells activation, and eventually improving ICI response. The synergic effect of targeting DDR1 with nilotinib and ICI with well tolerance is of appealing therapeutic value in HCC, especially those with advanced cirrhosis.

## Conclusion

Cirrhotic-ECM featured by Col1 enrichment impairs the response of HCC to ICI by orchestrating a neutrophils/NETs-dominant immune-suppressive TME through DDR1-NFκB-CXCL8 axis. This provides a mechanical insight, as well as a potential combination strategy by targeting DDR1 to enhance ICI response in HCC patients with cirrhosis (Fig. 6N).

### Supplementary Information

**Additional file 1: Figure S1.** ECM gene expression in HCC. **A** Heatmap showing the ECM gene expression. **B** ECM score distribution between HCC with or without cirrhosis. **C** Kaplan–Meier plot showing the OS difference between Matrix score high HCC and matrix score low HCC.

**Additional file 2: Figure S2.** Col1 deposition is related with suppressed T cell cytotoxicity. **A**, **B** IHC showed CD8+ T cell density, GZMB and TNFA intensity in subcutaneous Hepa1-6 tumor with or without intra-tumor Col1 deposition. Scale Bar: 100 μm.

**Additional file 3: Figure S3.** Scheme plot showed the analysis procedure comparing the biological difference between matrix score high and matrix score low HCC.

**Additional file 4: Figure S4.** Microenvironment difference between ECM score high and low HCC. **A** Protein abundance difference of neutrophil marker MPO and T cell marker in para-tumor and tumor tissue between HCC stratified with top 25%, top 50%, top 75% and bottom 25% ECM score in CHCC-HBV cohort. **B** Cell fraction difference between ECM score high and low HCC. **C**, **D** NETs gene expression difference and GSEA analysis outcome between ECM score high and low HCC.

**Additional file 5: Figure S5.** T cell and neutrophil enrichment difference between HCC with S4 liver fibrosis compared to S0/S1 fibrosis. **A** Representative TSNE plot of T cell and neutrophil enrichment among CD45+ immune cell in HCC microenvironment detected by flow cytometry. **B** Gate strategy of neutrophil detection. **C** Boxplot showed T cell and neutrophil difference between HCC with S4 liver fibrosis (n = 12) compared to S0/S1 cirrhosis (n = 13).

**Additional file 6: Figure S6.** DMN-induced fibrosis increased neutrophil infiltration in HDI-induced HCC model. **A** Model establishment scheme of HDI-induced HCC model with DMN-induced cirrhosis background. **B**, **C** Representative tumor image of HDI-induced HCC model and orthotopic HCC model. **D** Representative IHC image of CD8+ T cell (CD8) and neutrophil (MPO) of HDI-induced HCC tumor with or without DMN-induced cirrhosis background. Scale Bar: 50 μm.

**Additional file 7: Figure S7.** Col1 did not increase chemotaxis of neutrophils.

**Additional file 8: Figure S8.** Representative image of the spatial distribution of T cell (red) NETs (green) and HepG2 cell (blue) as target. NETs provoking or inhibition was carried by 1 μg/ml CXCL8 or 5 μg/ml CXCR2 inhibitor administration.

**Additional file 9: Figure S9.** Cytometer measurement of IFN-γ and GZMB in CD8+ T cell co-cultured with HepG2 with or without NETs.

**Additional file 10: Figure S10.** Correlation of p65, DDR1, CXCL8 and FUT4 in TCGA-LIHC cohort.

**Additional file 11: Figure S11.** DDR1-NFκB upregulates CXCL8 in HCC cell line. **A** WB analysis the expression level alteration of p-p65 and CXCL8 after DDR1 knockdown or overexpression in HepG2 cell line. **B** Quantification of the mRNA level alteration of CXCL8 in HepG2 cell line after DDR1 knockdown or overexpression, or co-cultured with Col1, by qPCR. **C** Western Blot analysis the expression level alteration of DDR1, pDDR1, p65, p-p65 and CXCL8 after co-culture with Col1 in HepG2 and PLC/PRF/5 cell line.

**Additional file 12: Figure S12.** Regular blood test, hepatic and nephrotic function test in mice received aPD-1, aPD-1 + nilotinib and nilotinib treatment

**Additional file 13: Table S1.** Clinical character of HCC with different liver fibrosis stage.

**Additional file 14: Table S2.** Genes used to establish ECM score.

**Additional file 15: Table S3.** Primer sequence used in this study.

**Additional file 16: Table S4.** NanoLC-MS/MS proteomic analysis of cirrhotic-ECM.

**Additional file 17: Table S5.** RNAseq analysis of HCC cell line stimulated with cirrhotic-ECM.

## Data Availability

The datasets in the current study are available from the corresponding author on reasonable request.

## Abbreviations

HCC: Hepatocellular carcinoma
ICI: Immune checkpoint inhibitor
ECM: Extracellular matrix
Col1: Collagen type I
NETs: Neutrophil extracellular traps
aPD-1: Anti PD-1
nanoLC-MS/MS: Nanoscale liquid chromatography coupled to tandem mass spectrometry
IHC: Immunohistochemical
IF: Immunofluorescence

## References

[1] Sung H, Ferlay J, Siegel RL, Laversanne M, Soerjomataram I, Jemal A (2021). Global cancer statistics 2020: GLOBOCAN estimates of incidence and mortality worldwide for 36 cancers in 185 countries. CA Cancer J Clin.

[2] Sangro B, Sarobe P, Hervas-Stubbs S, Melero I (2021). Advances in immunotherapy for hepatocellular carcinoma. Nat Rev Gastroenterol Hepatol.

[3] Bruix J, Chan SL, Galle PR, Rimassa L, Sangro B (2021). Systemic treatment of hepatocellular carcinoma: an EASL position paper. J Hepatol.

[4] Piersma B, Hayward MK, Weaver VM (2020). Fibrosis and cancer: a strained relationship. Biochim Biophys Acta Rev Cancer.

[5] Kalaitzakis E, Gunnarsdottir SA, Josefsson A, Bjornsson E (2011). Increased risk for malignant neoplasms among patients with cirrhosis. Clin Gastroenterol Hepatol.

[6] Keenan BP, Fong L, Kelley RK (2019). Immunotherapy in hepatocellular carcinoma: the complex interface between inflammation, fibrosis, and the immune response. J Immunother Cancer.

[7] Mushtaq MU, Papadas A, Pagenkopf A, Flietner E, Morrow Z, Chaudhary SG (2018). Tumor matrix remodeling and novel immunotherapies: the promise of matrix-derived immune biomarkers. J Immunother Cancer.

[8] Nicolas-Boluda A, Vaquero J, Vimeux L, Guilbert T, Barrin S, Kantari-Mimoun C (2021). Tumor stiffening reversion through collagen crosslinking inhibition improves T cell migration and anti-PD-1 treatment. Elife.

[9] Jung BK, Ko HY, Kang H, Hong J, Ahn HM, Na Y (2020). Relaxin-expressing oncolytic adenovirus induces remodeling of physical and immunological aspects of cold tumor to potentiate PD-1 blockade. J Immunother Cancer.

[10] Hedrick CC, Malanchi I (2022). Neutrophils in cancer: heterogeneous and multifaceted. Nat Rev Immunol.

[11] Xue R, Zhang Q, Cao Q, Kong R, Xiang X, Liu H (2022). Liver tumour immune microenvironment subtypes and neutrophil heterogeneity. Nature.

[12] Yang LY, Luo Q, Lu L, Zhu WW, Sun HT, Wei R (2020). Increased neutrophil extracellular traps promote metastasis potential of hepatocellular carcinoma via provoking tumorous inflammatory response. J Hematol Oncol.

[13] Teijeira A, Garasa S, Gato M, Alfaro C, Migueliz I, Cirella A (2020). CXCR1 and CXCR2 chemokine receptor agonists produced by tumors induce neutrophil extracellular traps that interfere with immune cytotoxicity. Immunity.

[14] Teijeira A, Garasa S, Ochoa MC, Villalba M, Olivera I, Cirella A (2021). IL8, neutrophils, and NETs in a collusion against cancer immunity and immunotherapy. Clin Cancer Res.

[15] Zhang Y, Chandra V, Riquelme Sanchez E, Dutta P, Quesada PR, Rakoski A (2020). Interleukin-17-induced neutrophil extracellular traps mediate resistance to checkpoint blockade in pancreatic cancer. J Exp Med.

[16] Gao Q, Zhu H, Dong L, Shi W, Chen R, Song Z (2019). Integrated proteogenomic characterization of HBV-related hepatocellular carcinoma. Cell.

[17] Sun R, Zhang Z, Bao R, Guo X, Gu Y, Yang W (2022). Loss of SIRT5 promotes bile acid-induced immunosuppressive microenvironment and hepatocarcinogenesis. J Hepatol.

[18] Shao X, Taha IN, Clauser KR, Gao YT, Naba A (2020). MatrisomeDB: the ECM-protein knowledge database. Nucleic Acids Res.

[19] Chakravarthy A, Khan L, Bensler NP, Bose P, De Carvalho DD (2018). TGF-beta-associated extracellular matrix genes link cancer-associated fibroblasts to immune evasion and immunotherapy failure. Nat Commun.

[20] Shen XT, Xie SZ, Xu J, Yang LY, Qin LX (2022). Pan-cancer analysis reveals a distinct neutrophil extracellular trap-associated regulatory pattern. Front Immunol.

[21] Chen J, Lin Z, Liu L, Zhang R, Geng Y, Fan M (2021). GOLM1 exacerbates CD8(+) T cell suppression in hepatocellular carcinoma by promoting exosomal PD-L1 transport into tumor-associated macrophages. Signal Transduct Target Ther.

[22] Hartmann N, Giese NA, Giese T, Poschke I, Offringa R, Werner J (2014). Prevailing role of contact guidance in intrastromal T-cell trapping in human pancreatic cancer. Clin Cancer Res.

[23] Salmon H, Franciszkiewicz K, Damotte D, Dieu-Nosjean MC, Validire P, Trautmann A (2012). Matrix architecture defines the preferential localization and migration of T cells into the stroma of human lung tumors. J Clin Invest.

[24] Chetoui N, El Azreq MA, Boisvert M, Bergeron ME, Aoudjit F (2011). Discoidin domain receptor 1 expression in activated T cells is regulated by the ERK MAP kinase signaling pathway. J Cell Biochem.

[25] Vijver SV, Singh A, Mommers-Elshof E, Meeldijk J, Copeland R, Boon L (2021). Collagen fragments produced in cancer mediate T cell suppression through leukocyte-associated immunoglobulin-like receptor 1. Front Immunol.

[26] Peng DH, Rodriguez BL, Diao L, Chen L, Wang J, Byers LA (2020). Collagen promotes anti-PD-1/PD-L1 resistance in cancer through LAIR1-dependent CD8(+) T cell exhaustion. Nat Commun.

[27] Sun X, Wu B, Chiang HC, Deng H, Zhang X, Xiong W (2021). Tumour DDR1 promotes collagen fibre alignment to instigate immune exclusion. Nature.

[28] Hammerl D, Martens JWM, Timmermans M, Smid M, Trapman-Jansen AM, Foekens R (2021). Spatial immunophenotypes predict response to anti-PD1 treatment and capture distinct paths of T cell evasion in triple negative breast cancer. Nat Commun.

[29] Kaltenmeier C, Yazdani HO, Morder K, Geller DA, Simmons RL, Tohme S (2021). Neutrophil extracellular traps promote T cell exhaustion in the tumor microenvironment. Front Immunol.

[30] Schauer C, Janko C, Munoz LE, Zhao Y, Kienhofer D, Frey B (2014). Aggregated neutrophil extracellular traps limit inflammation by degrading cytokines and chemokines. Nat Med.

[31] Chen Y, Yang S, Tavormina J, Tampe D, Zeisberg M, Wang H (2022). Oncogenic collagen I homotrimers from cancer cells bind to alpha3beta1 integrin and impact tumor microbiome and immunity to promote pancreatic cancer. Cancer Cell.

[32] Romer AMA, Thorseth ML, Madsen DH (2021). Immune modulatory properties of collagen in cancer. Front Immunol.

[33] Filliol A, Saito Y, Nair A, Dapito DH, Yu LX, Ravichandra A (2022). Opposing roles of hepatic stellate cell subpopulations in hepatocarcinogenesis. Nature.

[34] Deng J, Kang Y, Cheng CC, Li X, Dai B, Katz MH (2021). DDR1-induced neutrophil extracellular traps drive pancreatic cancer metastasis. JCI Insight.

[35] Su H, Yang F, Fu R, Trinh B, Sun N, Liu J (2022). Collagenolysis-dependent DDR1 signalling dictates pancreatic cancer outcome. Nature.

[36] Elkamhawy A, Lu Q, Nada H, Woo J, Quan G, Lee K (2021). The journey of DDR1 and DDR2 kinase inhibitors as rising stars in the fight against cancer. Int J Mol Sci.

[37] Duan X, Xu X, Zhang Y, Gao Y, Zhou J, Li J (2022). DDR1 functions as an immune negative factor in colorectal cancer by regulating tumor-infiltrating T cells through IL-18. Cancer Sci.

[38] Plosker GL, Robinson DM (2008). Nilotinib. Drugs.

[39] Nokin MJ, Darbo E, Travert C, Drogat B, Lacouture A, San Jose S (2020). Inhibition of DDR1 enhances in vivo chemosensitivity in KRAS-mutant lung adenocarcinoma. JCI Insight.

