# Supplementary figures and images for: Cirrhotic-extracellular matrix attenuates aPD-1 treatment response by initiating immunosuppressive neutrophil extracellular traps formation in hepatocellular carcinoma
Source: Exp Hematol Oncol. 2024 Feb 22;13:20. doi: 10.1186/s40164-024-00476-9 (PMC10882882; doi:10.1186/s40164-024-00476-9)

A

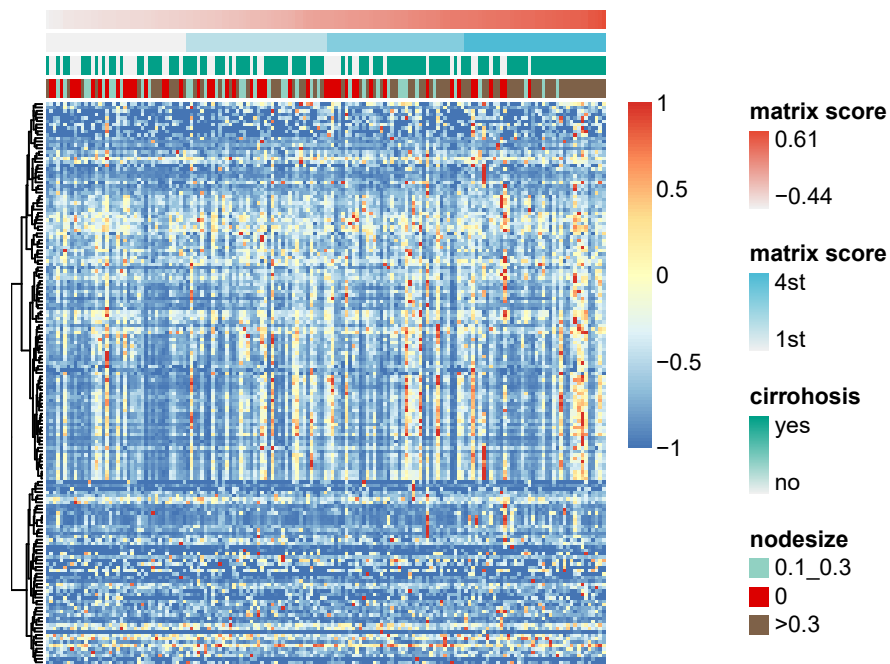

B

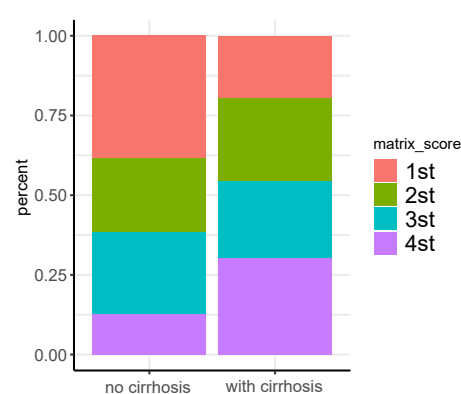

C

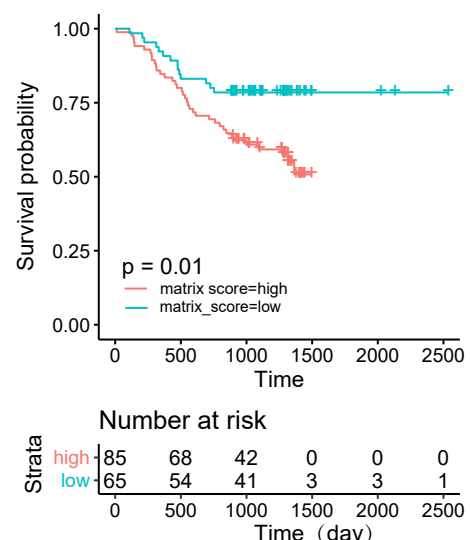

Supplement: Supplementary file 1 — Additional file 1: Figure S1. ECM gene expression in HCC. A Heatmap showing the ECM gene expression. B ECM score distribution between HCC with or without cirrhosis. C Kaplan–Meier plot showing the OS difference between Matrix score high HCC and matrix score low HCC. [file 40164_2024_476_MOESM1_ESM.pdf]

A

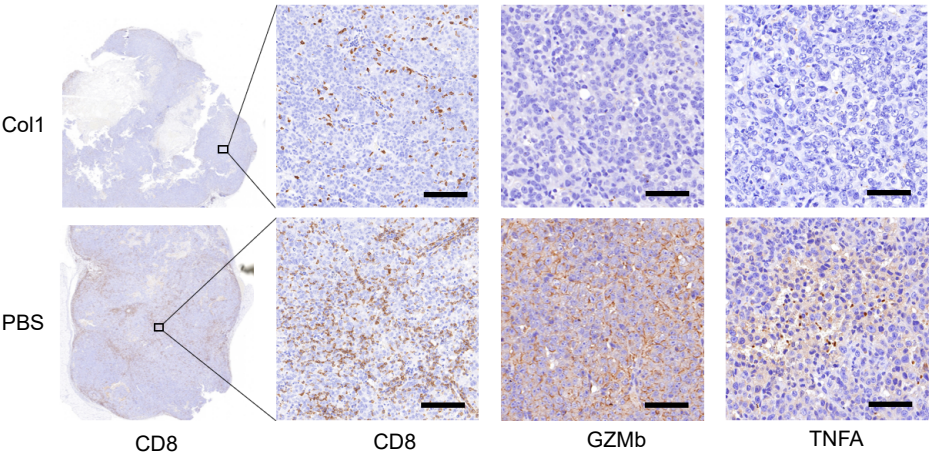

B

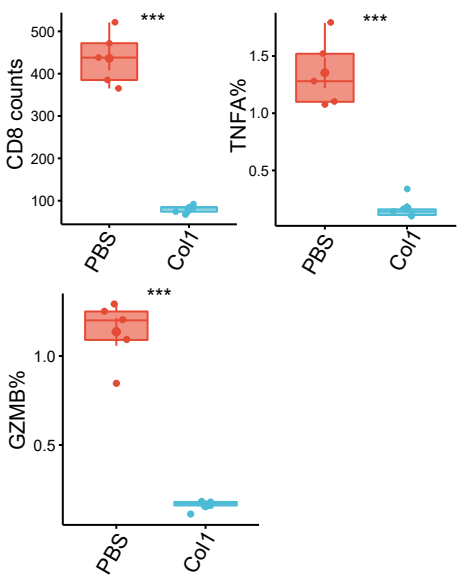

Supplement: Supplementary file 2 — Additional file 2: Figure S2. Col1 deposition is related with suppressed T cell cytotoxicity. A, B IHC showed CD8+ T cell density, GZMB and TNFA intensity in subcutaneous Hepa1-6 tumor with or without intra-tumor Col1 deposition. Scale Bar: 100 μm. [file 40164_2024_476_MOESM2_ESM.pdf]

A

ECM\_score low

ECM\_score high

different expressed gene

annotation

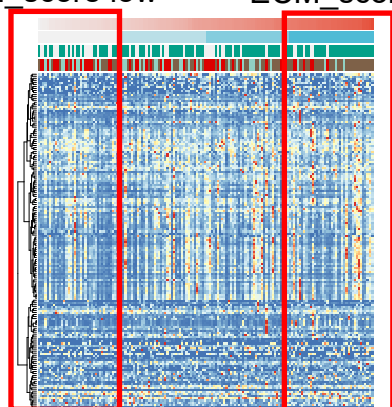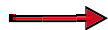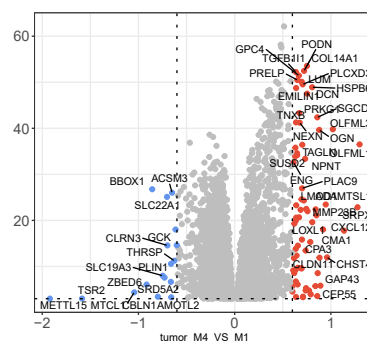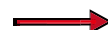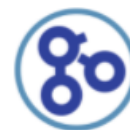

Supplement: Supplementary file 3 — Additional file 3: Figure S3. Scheme plot showed the analysis procedure comparing the biological difference between matrix score high and matrix score low HCC. [file 40164_2024_476_MOESM3_ESM.pdf]

A

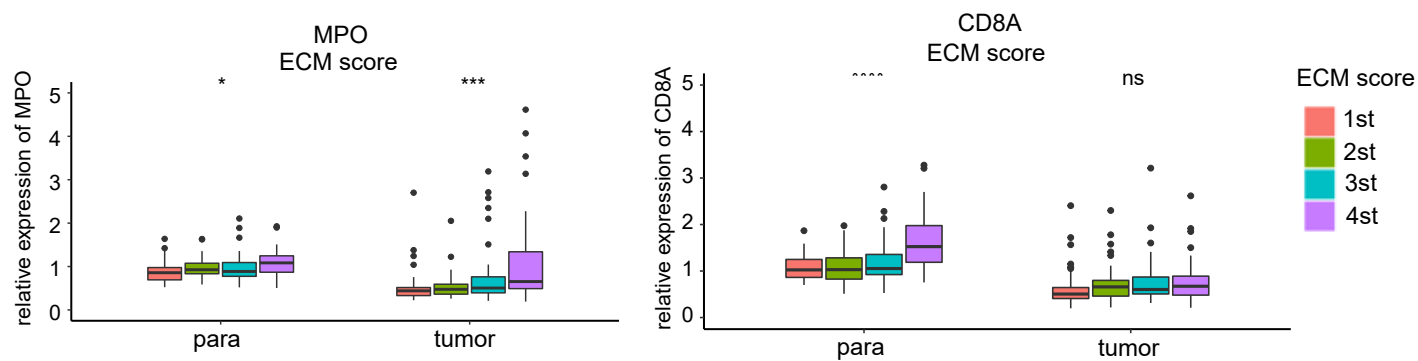

B

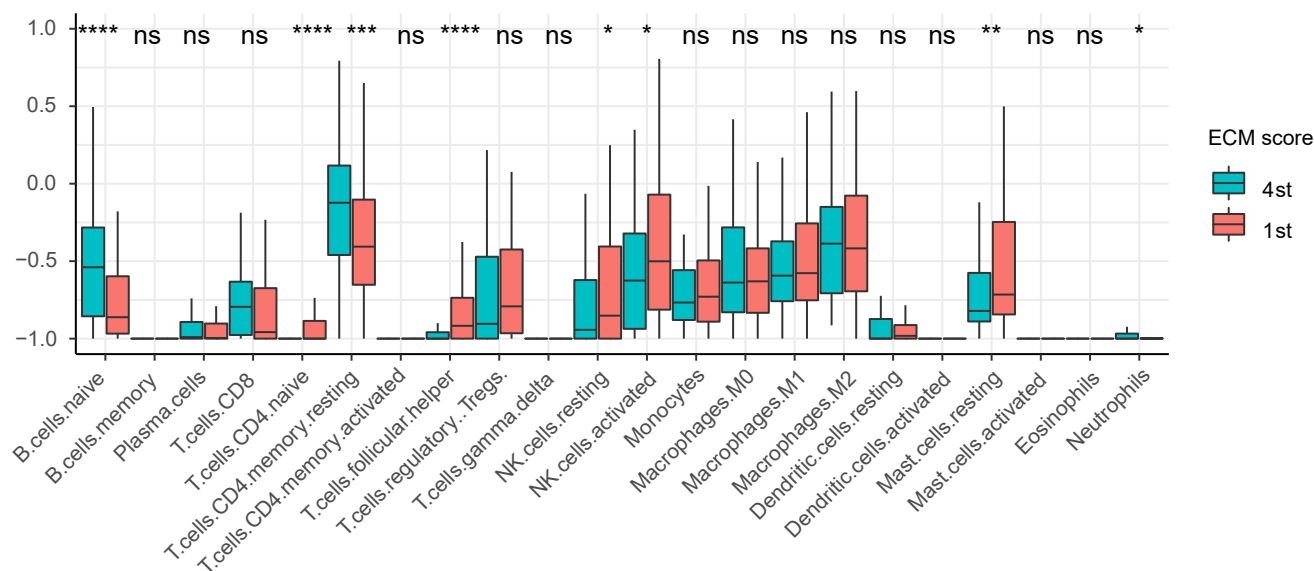

C

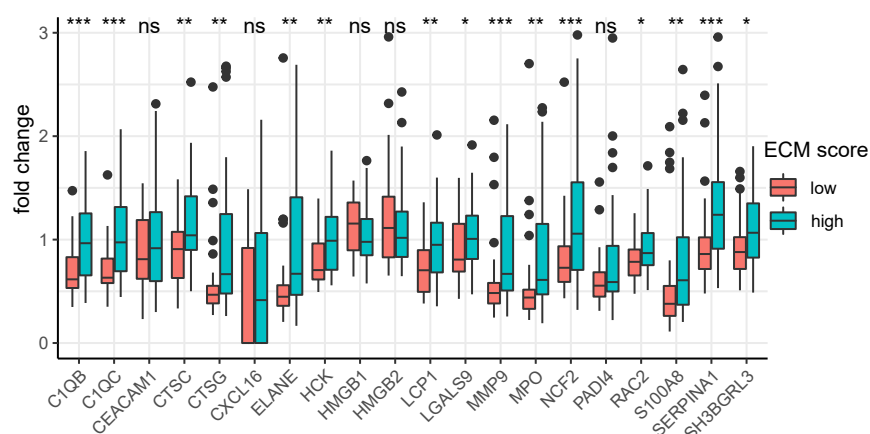

D

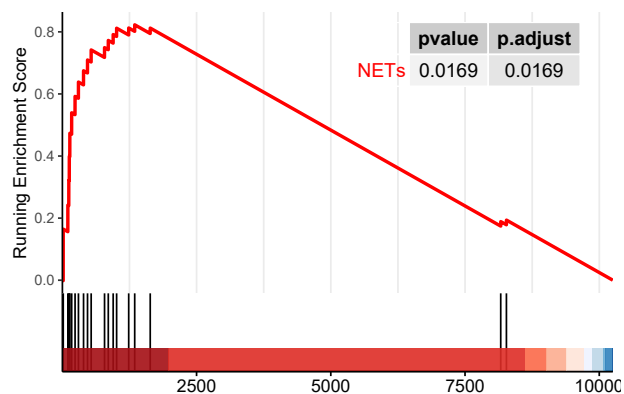

Supplement: Supplementary file 4 — Additional file 4: Figure S4. Microenvironment difference between ECM score high and low HCC. A Protein abundance difference of neutrophil marker MPO and T cell marker in para-tumor and tumor tissue between HCC stratified with top 25%, top 50%, top 75% and bottom 25% ECM score in CHCC-HBV cohort. B Cell fraction difference between ECM score high and low HCC. C, D NETs gene expression difference and GSEA analysis outcome between ECM score high and low HCC. [file 40164_2024_476_MOESM4_ESM.pdf]

A

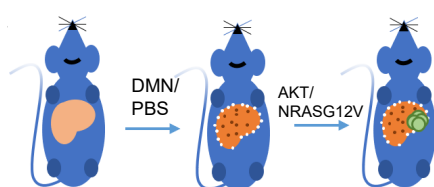

B

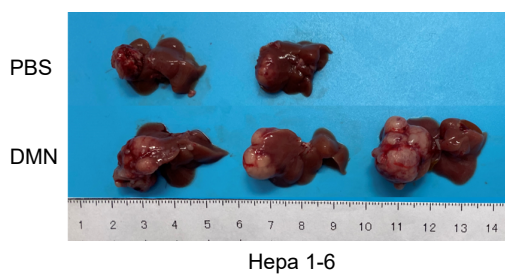

C

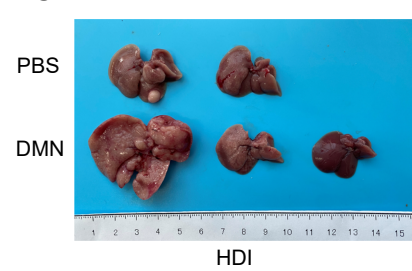

D

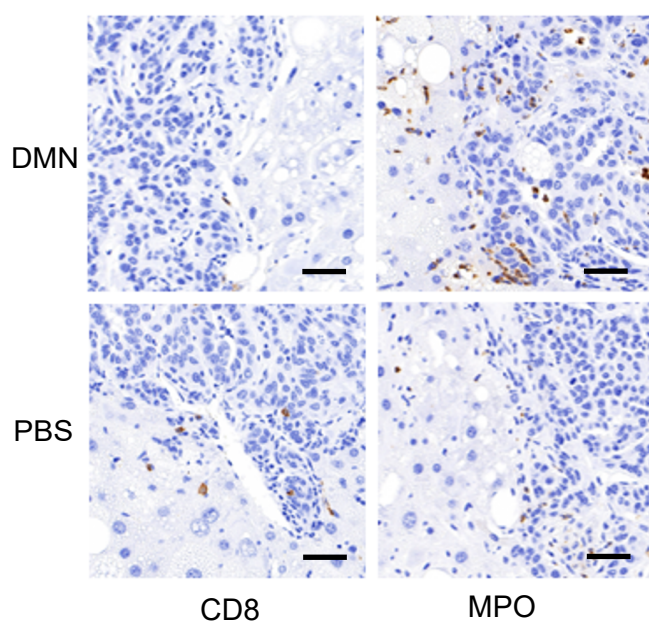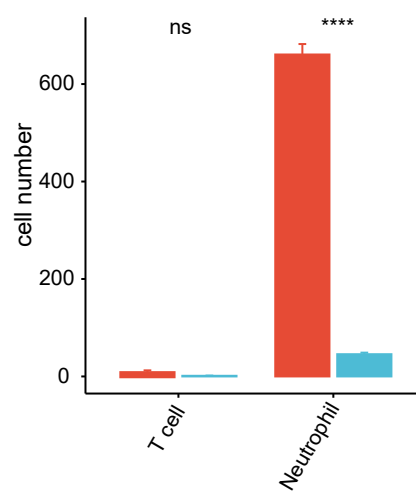

Supplement: Supplementary file 6 — Additional file 6: Figure S6. DMN-induced fibrosis increased neutrophil infiltration in HDI-induced HCC model. A Model establishment scheme of HDI-induced HCC model with DMN-induced cirrhosis background. B, C Representative tumor image of HDI-induced HCC model and orthotopic HCC model. D Representative IHC image of CD8+ T cell (CD8) and neutrophil (MPO) of HDI-induced HCC tumor with or without DMN-induced cirrhosis background. Scale Bar: 50 μm. [file 40164_2024_476_MOESM6_ESM.pdf]

A

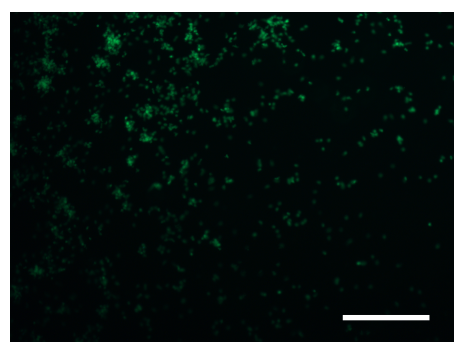

Col1

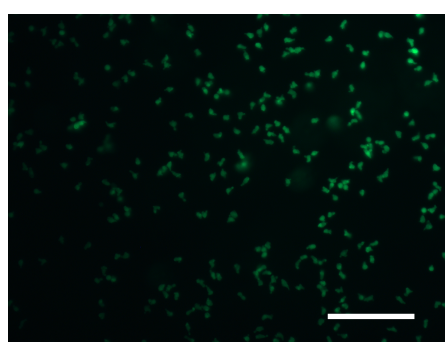

PBS

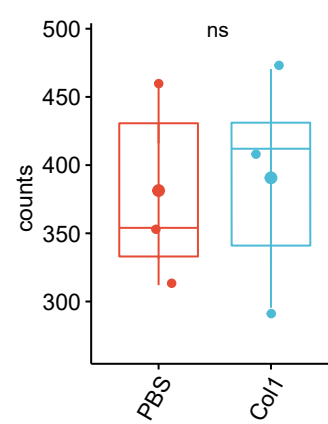

Supplement: Supplementary file 7 — Additional file 7: Figure S7. Col1 did not increase chemotaxis of neutrophils. [file 40164_2024_476_MOESM7_ESM.pdf]

HepG2  
NETs  
T cell

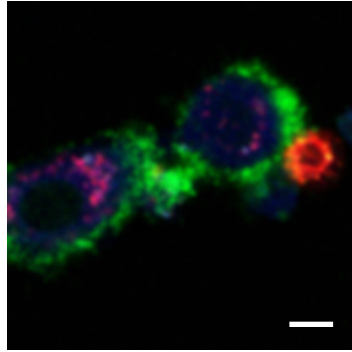

CXCL8

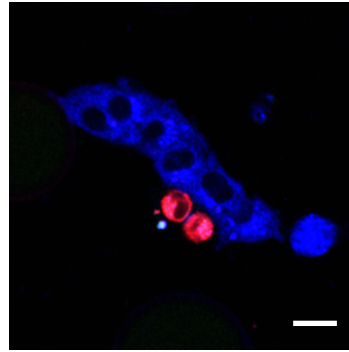

CXCL8+CXCR2in

Supplement: Supplementary file 8 — Additional file 8: Figure S8. Representative image of the spatial distribution of T cell (red) NETs (green) and HepG2 cell (blue) as target. NETs provoking or inhibition was carried by 1 μg/ml CXCL8 or 5 μg/ml CXCR2 inhibitor administration. [file 40164_2024_476_MOESM8_ESM.pdf]

G2+NETs+T cells

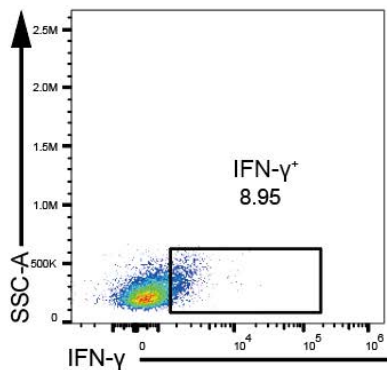

G2+T cells

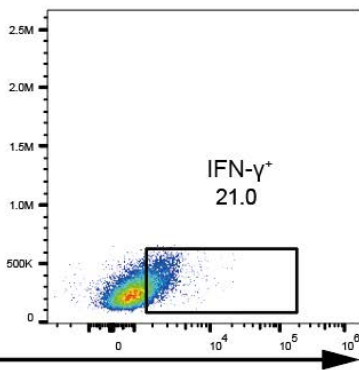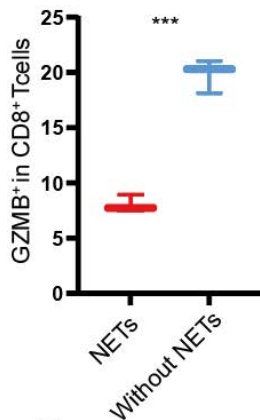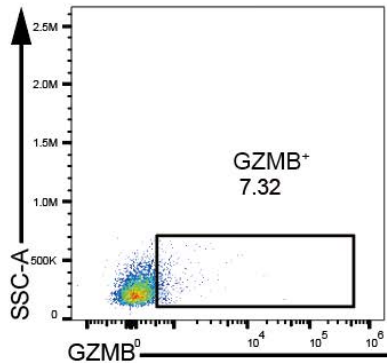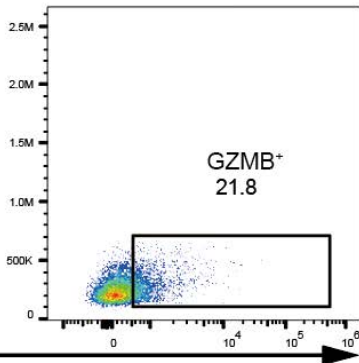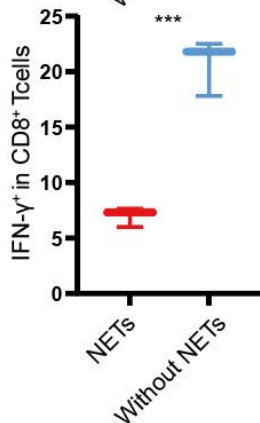

Supplement: Supplementary file 9 — Additional file 9: Figure S9. Cytometer measurement of IFN-γ and GZMB in CD8+ T cell co-cultured with HepG2 with or without NETs. [file 40164_2024_476_MOESM9_ESM.pdf]

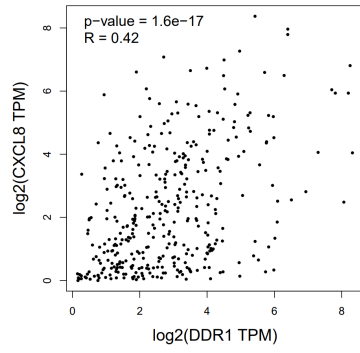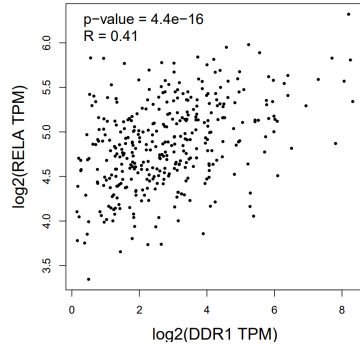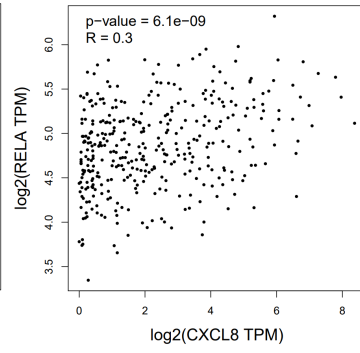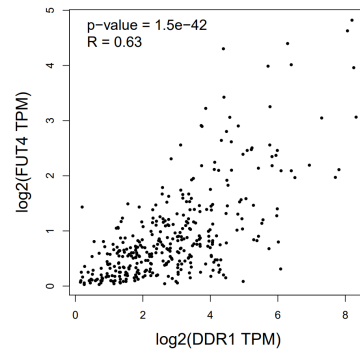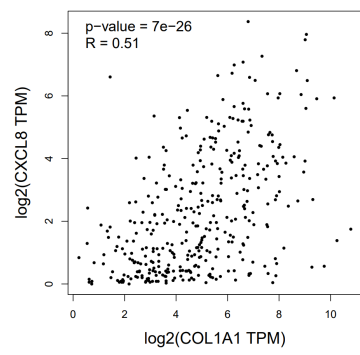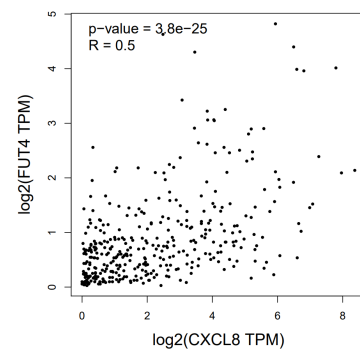

Supplement: Supplementary file 10 — Additional file 10: Figure S10. Correlation of p65, DDR1, CXCL8 and FUT4 in TCGA-LIHC cohort. [file 40164_2024_476_MOESM10_ESM.pdf]

A

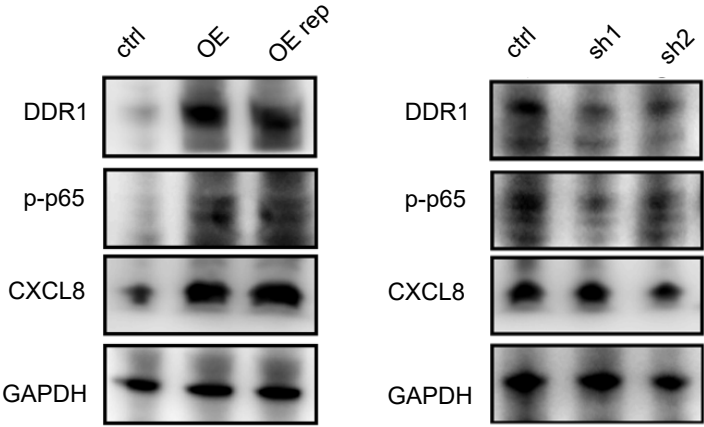

B

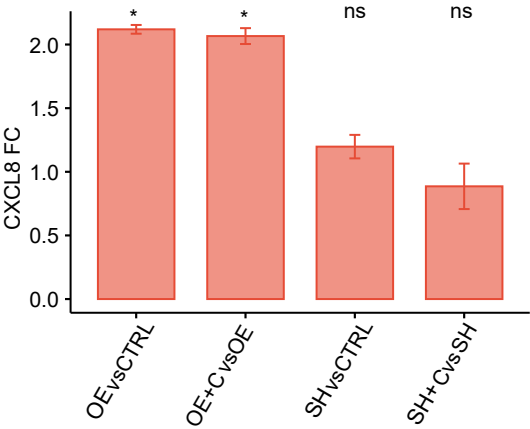

HepG2

C

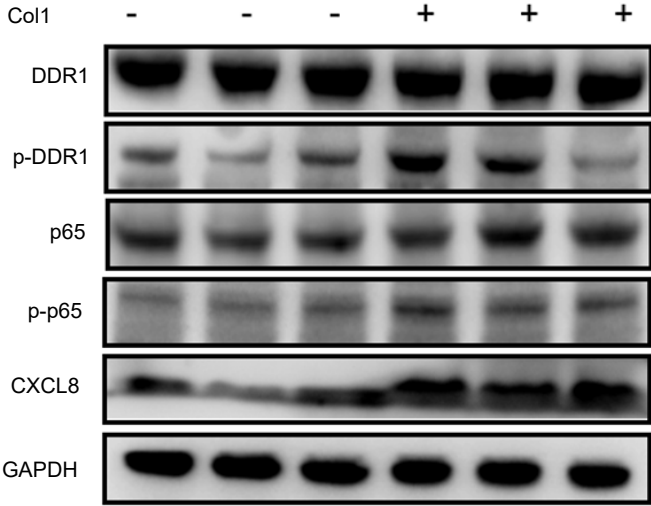

HepG2

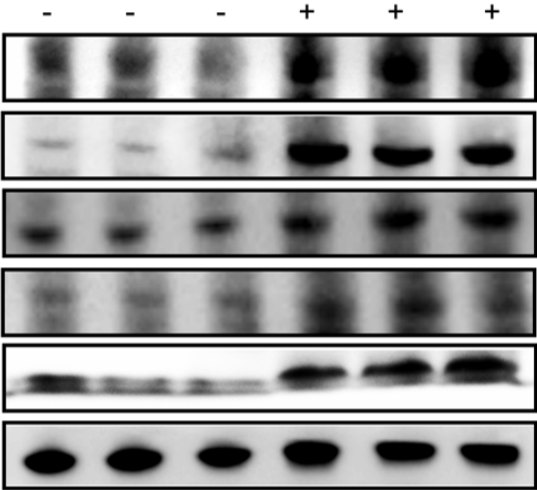

PLC

Supplement: Supplementary file 11 — Additional file 11: Figure S11. DDR1-NFκB upregulates CXCL8 in HCC cell line. A WB analysis the expression level alteration of p-p65 and CXCL8 after DDR1 knockdown or overexpression in HepG2 cell line. B Quantification of the mRNA level alteration of CXCL8 in HepG2 cell line after DDR1 knockdown or overexpression, or co-cultured with Col1, by qPCR. C Western Blot analysis the expression level alteration of DDR1, pDDR1, p65, p-p65 and CXCL8 after co-culture with Col1 in HepG2 and PLC/PRF/5 cell line. [file 40164_2024_476_MOESM11_ESM.pdf]

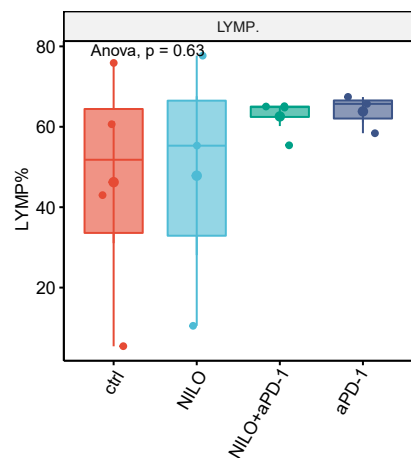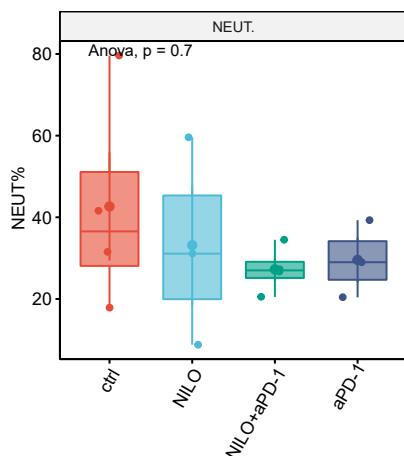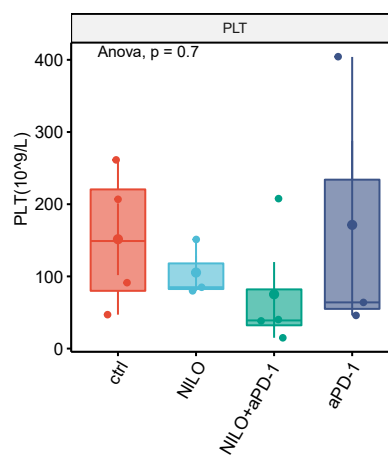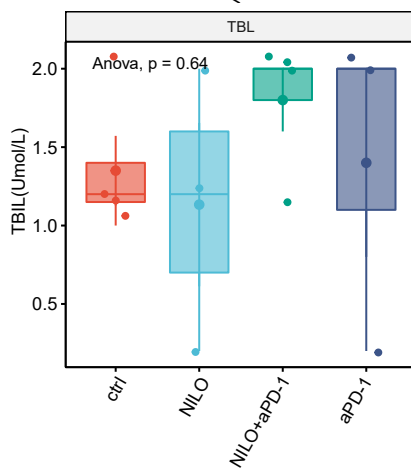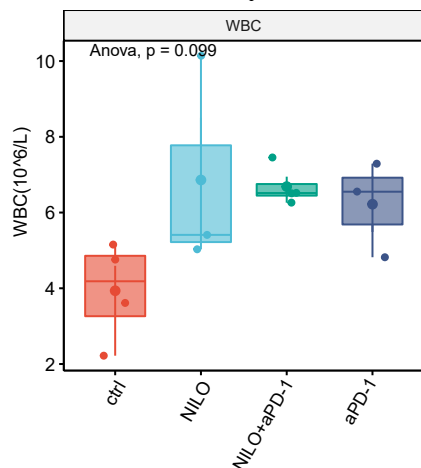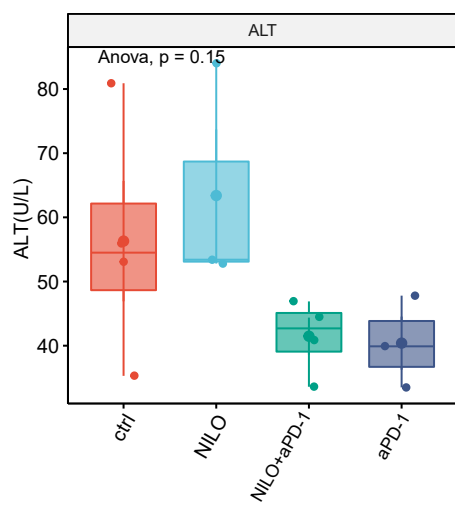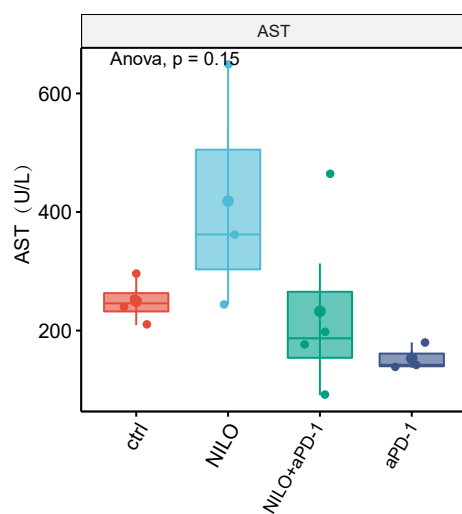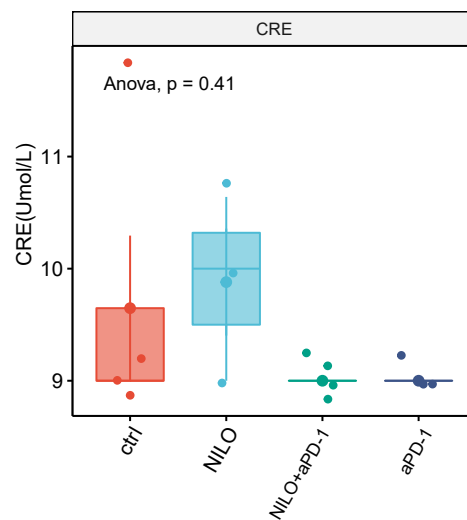

Supplement: Supplementary file 12 — Additional file 12: Figure S12. Regular blood test, hepatic and nephrotic function test in mice received aPD-1, aPD-1 + nilotinib and nilotinib treatment [file 40164_2024_476_MOESM12_ESM.pdf]
