# Supplementary material for: Cirrhotic-extracellular matrix attenuates aPD-1 treatment response by initiating immunosuppressive neutrophil extracellular traps formation in hepatocellular carcinoma
Source: Exp Hematol Oncol. 2024 Feb 22;13:20. doi: 10.1186/s40164-024-00476-9 (PMC10882882; doi:10.1186/s40164-024-00476-9)

A

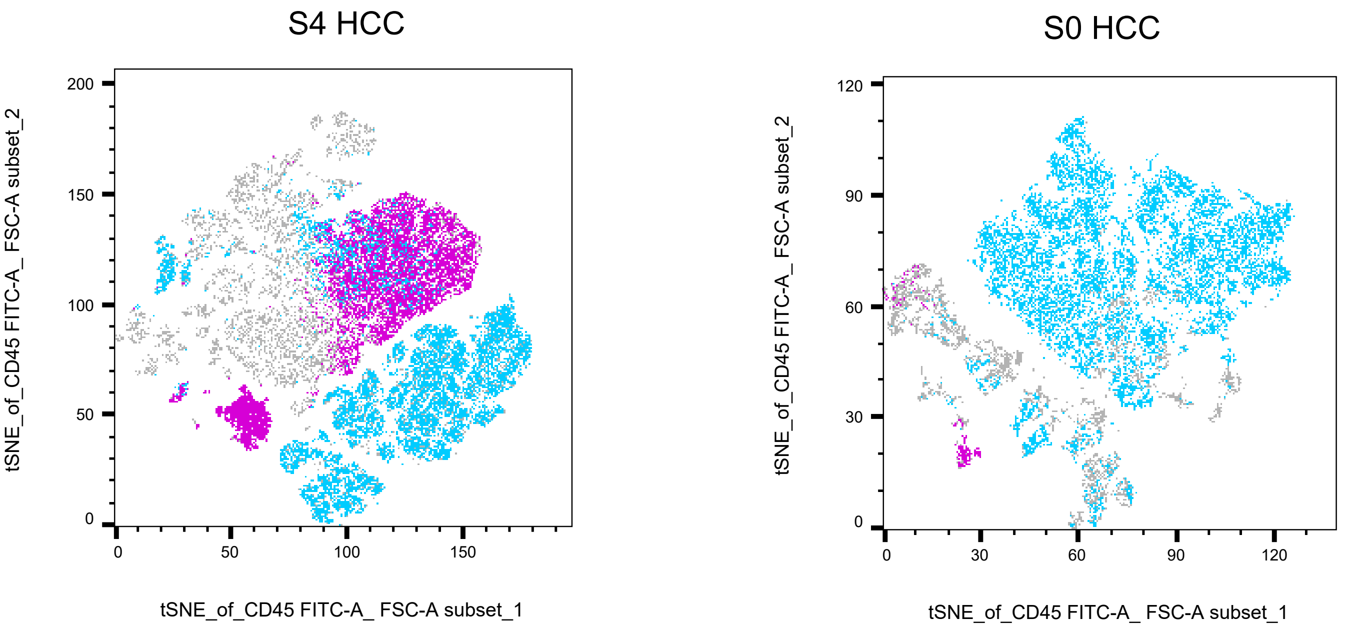

			. CD45 FITC-A, FSC-A subset
(cyan)	L07-G-PA1-1.fcs	T cell	36.4
(magenta)	L07-G-PA1-1.fcs	neutrophil	17.4
(grey)	L07-G-PA1-1.fcs	CD45 FITC-A, FSC-A subset	100

			. CD45 FITC-A, FSC-A subset
(cyan)	L26-PA1-1.fcs	T cell	71.9
(magenta)	L26-PA1-1.fcs	neutrophil	1.64
(grey)	L26-PA1-1.fcs	CD45 FITC-A, FSC-A subset	100

B

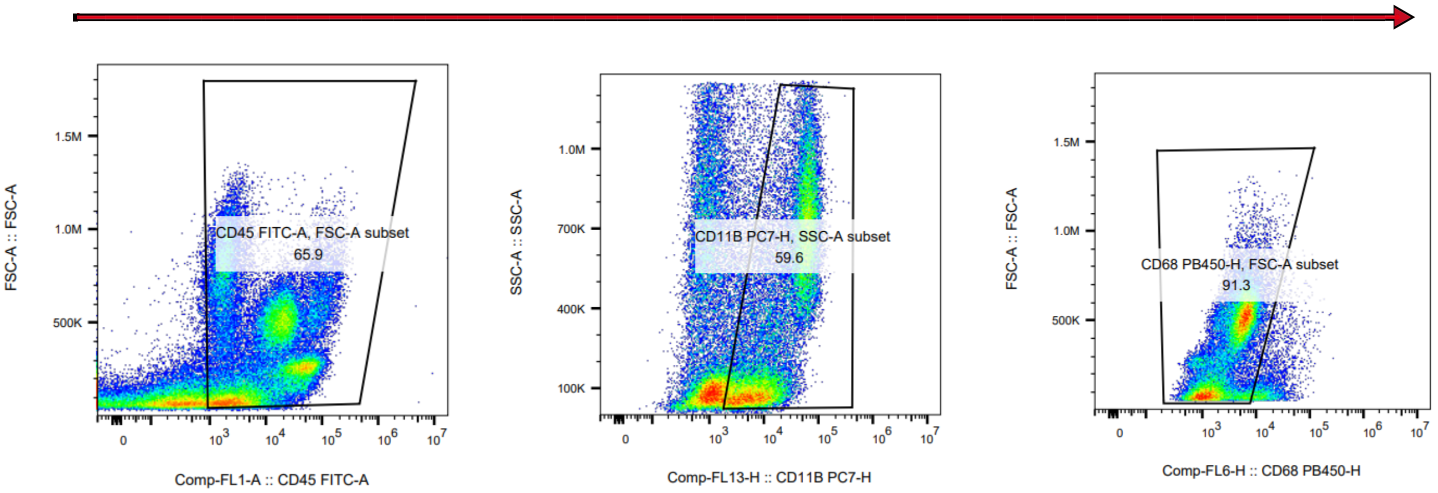

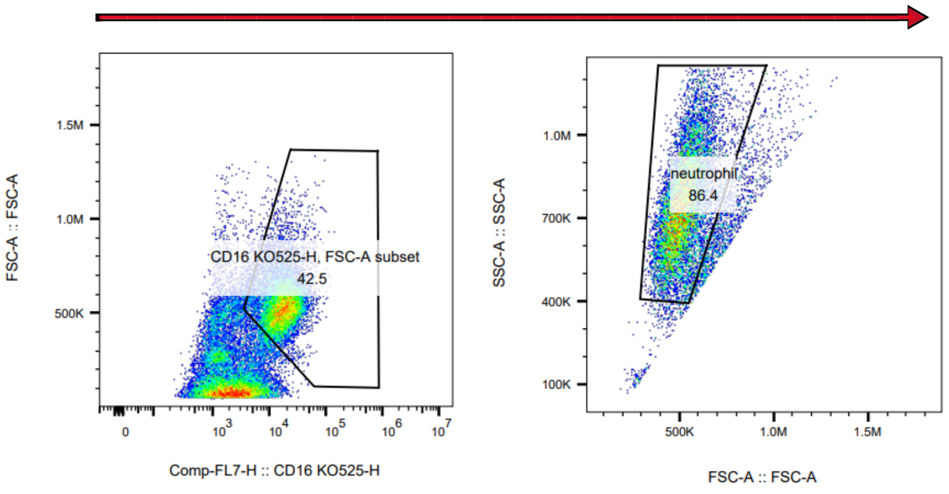

C

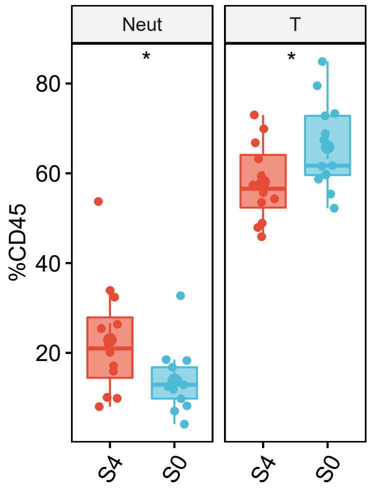

Supplement: Supplementary file 5 — Additional file 5: Figure S5. T cell and neutrophil enrichment difference between HCC with S4 liver fibrosis compared to S0/S1 fibrosis. A Representative TSNE plot of T cell and neutrophil enrichment among CD45+ immune cell in HCC microenvironment detected by flow cytometry. B Gate strategy of neutrophil detection. C Boxplot showed T cell and neutrophil difference between HCC with S4 liver fibrosis (n = 12) compared to S0/S1 cirrhosis (n = 13). [file 40164_2024_476_MOESM5_ESM.pdf]
